# Sex-specific molecular signature of mouse podocytes in homeostasis and in response to pharmacological challenge with rapamycin

**DOI:** 10.1186/s13293-024-00647-7

**Published:** 2024-09-15

**Authors:** Ola Al-Diab, Christin Sünkel, Eric Blanc, Rusan Ali Catar, Muhammad Imtiaz Ashraf, Hongfan Zhao, Pinchao Wang, Markus M. Rinschen, Raphaela Fritsche-Guenther, Florian Grahammer, Sebastian Bachmann, Dieter Beule, Jennifer A. Kirwan, Nikolaus Rajewsky, Tobias B. Huber, Dennis Gürgen, Angelika Kusch

**Affiliations:** 1 Department of Nephrology and Medical Intensive Care, Charité-Universitätsmedizin Berlin, corporate member of Freie Universität Berlin and Humboldt-Universität zu Berlin, Charitéplatz 1, 10117 Berlin, Germany; 2 Berlin Institute for Medical Systems Biology (BIMSB), Max Delbrück Center for Molecular Medicine in the Helmholtz Association, Hannoversche Str 28, 10115 Berlin, Germany; 3 Core Unit Bioinformatics, Berlin Institute of Health at Charité – Universitätsmedizin Berlin, Charitéplatz 1, 10117 Berlin, Germany; 4 Department of Surgery, Experimental Surgery, Charité-Universitätsmedizin Berlin, corporate member of Freie Universität Berlin and Humboldt-Universität zu Berlin, Augustenburger Platz 1, 13353 Berlin, Germany; 5 Department of Urology, Xijing Hospital, Fourth Military Medical University, Xi’an, Shaanxi China; 6 III. Department of Medicine, University Hospital Hamburg Eppendorf, Martinistraße 52, 20246 Hamburg, Germany; 7 Metabolomics Platform, Berlin Institute of Health at Charité – Universitätsmedizin Berlin, Lindenberger Weg 80, 10117 Berlin, Germany; 8 Institute of Functional Anatomy, Charité-Universitätsmedizin Berlin, corporate member of Freie Universität Berlin and Humboldt-Universität zu Berlin, Charitéplatz 1, 10117 Berlin, Germany; 9 BIH Biomedical Innovation Academy (BIA), Berlin Institute of Health at Charité – Universitätsmedizin Berlin, Charitéplatz 1, 10117 Berlin, Germany; 10 Department of Biomedicine, Aarhus University, Aarhus, Denmark; 11 Department of Pediatric Oncology and Hematology, Charité-Universitätsmedizin Berlin, corporate member of Freie Universität Berlin and Humboldt-Universität zu Berlin, Augustenburger Platz 1, 13353 Berlin, Germany; 12 Experimental Pharmacology & Oncology Berlin-Buch GmbH, 13125 Berlin-Buch, Germany

**Keywords:** Podocytes, Sex differences, Transcriptome, Proteome, Metabolome, mTOR

## Abstract

**Background:**

Sex differences exist in the prevalence and progression of major glomerular diseases. Podocytes are the essential cell-type in the kidney which maintain the physiological blood-urine barrier, and pathological changes in podocyte homeostasis are critical accelerators of impairment of kidney function. However, sex-specific molecular signatures of podocytes under physiological and stress conditions remain unknown. This work aimed at identifying sexual dimorphic molecular signatures of podocytes under physiological condition and pharmacologically challenged homeostasis with mechanistic target of rapamycin (mTOR) inhibition. mTOR is a crucial regulator involved in a variety of physiological and pathological stress responses in the kidney and inhibition of this pathway may therefore serve as a general stress challenger to get fundamental insights into sex differences in podocytes.

**Methods:**

The genomic ROSAmT/mG-NPHS2 Cre mouse model was used which allows obtaining highly pure podocyte fractions for cell-specific molecular analyses, and vehicle or pharmacologic treatment with the mTOR inhibitor rapamycin was performed for 3 weeks. Subsequently, deep RNA sequencing and proteomics were performed of the isolated podocytes to identify intrinsic sex differences. Studies were supplemented with metabolomics from kidney cortex tissues.

**Results:**

Although kidney function and morphology remained normal in all experimental groups, RNA sequencing, proteomics and metabolomics revealed strong intrinsic sex differences in the expression levels of mitochondrial, translation and structural transcripts, protein abundances and regulation of metabolic pathways. Interestingly, rapamycin abolished prominent sex-specific clustering of podocyte gene expression and induced major changes only in male transcriptome. Several sex-biased transcription factors could be identified as possible upstream regulators of these sexually dimorphic responses. Concordant to transcriptomics, metabolomic changes were more prominent in males. Remarkably, high number of previously reported kidney disease genes showed intrinsic sexual dimorphism and/or different response patterns towards mTOR inhibition.

**Conclusions:**

Our results highlight remarkable intrinsic sex-differences and sex-specific response patterns towards pharmacological challenged podocyte homeostasis which might fundamentally contribute to sex differences in kidney disease susceptibilities and progression. This work provides rationale and an in-depth database for novel targets to be tested in specific kidney disease models to advance with sex-specific treatment strategies.

**Supplementary Information:**

The online version contains supplementary material available at 10.1186/s13293-024-00647-7.

## Plain Language Summary

The global burden of chronic kidney diseases is rapidly increasing and is projected to become the fifth most common cause of years of life lost worldwide by 2040. Sexual dimorphism in kidney diseases and transplantation is well known, yet sex-specific therapeutic strategies are still missing. One reason is the lack of knowledge due to the lack of inclusion of sex as a biological variable in study designs. This work aimed at identification of molecular signatures of male and female podocytes, gate-keepers of the glomerular filtration barrier. Like cardiomyocytes, podocytes are terminally differentiated cells which are highly susceptible towards pathological challenges. Podocytes are the decisive cell-type of the kidney to maintain the physiological blood-urine barrier, and disturbances of their homeostasis critically accelerate kidney function impairment. By help of a genomic mouse model, highly purified podocytes were obtained from male and female mice with and without pharmacological challenge of the mechanistic target of rapamycin (mTOR) signaling pathway which is known to be deregulated in major kidney diseases. Deep RNA sequencing, proteomics and metabolomics revealed strong intrinsic sex differences in the expression levels of mitochondrial, translation and structural transcripts, protein abundances and regulation of metabolic pathways which might fundamentally contribute to sex differences in kidney disease susceptibilities and progression. Remarkably, high number of previously reported kidney disease genes showed so far unknown intrinsic sexual dimorphism and/or different response patterns towards mTOR inhibition. Our work provides an in-depth database for novel targets to be tested in kidney disease models to advance with sex-specific treatment strategies.

**Supplementary Information:**

The online version contains supplementary material available at 10.1186/s13293-024-00647-7.

## Highlights

- First study to describe sexually dimorphic molecular signature of podocytes, “gate-keepers” of the kidney glomerular filtration barrier.
- Large number of known kidney-disease related genes are newly identified for intrinsic sexual dimorphic expression as well as after pharmacological intervention with rapamycin.
- Male podocytes appear more susceptible to transcriptomic changes.
- Metabolomics supports higher functional consequences of mTOR inhibition on protein synthesis and impaired energy balance in male kidneys.
- Study provides an in-depth database for further research in the field of highly sexually dimorphic kidney diseases.

**Supplementary Information:**

The online version contains supplementary material available at 10.1186/s13293-024-00647-7.

## Background

The maintenance of podocyte homeostasis critically determines kidney function during glomerular disease development and progression [1]. Podocytes are terminally differentiated cells of the glomerulus and represent the essential cell-type to maintain the integrity of the glomerular filtration barrier. A large body of evidence demonstrates sex differences in protein filtration and sex-different susceptibilities towards ischemic injury [2–5]. Male gender has been associated with more rapid progression and worse outcome in major chronic kidney diseases [6–9]. Sex differences in podocyte biology might play a decisive role in these processes. The need for addressing sex differences in studies of health-related research has been requested for more than two decades [10, 11]. Now, discussing sexual aspects has become statutory in large grant applications [12–15] and requirement in clinical studies [11]. Despite these regulatory efforts and the well-known sex differences in kidney physiology and pathology, research addressing this issue on the molecular level of podocytes remains sparse [16]. Therefore, this study was designed to achieve deeper insights into sexual dimorphism of the molecular signature of podocytes under normal homeostatic conditions and in response to inhibition of the mechanistic target of rapamycin (mTOR) signaling pathway with rapamycin.

The mTOR pathway is an important factor in the control of podocyte structure and function [17–19]. A well-balanced activation of this signaling pathway is crucial for compensatory mechanisms in diabetic nephropathy and focal segmental glomerulosclerosis [20]. In addition, mTOR inhibitors are commonly used as immunosuppressants [21]. Yet, it is still unknown why some kidney transplant patients develop de novo proteinuria while the majority of recipients of other solid organs improve their kidney function when on this medication. Furthermore, growing evidence suggests that mTOR inhibition improves physiological parameters associated with aging and aging-related disorders, thereby favoring longevity, interestingly to a higher extent in females compared to males [22–28]. We have recently reported sexual dimorphism in mTOR signaling in cardiomyocytes, with loss of cardioprotective phenotype in response to rapamycin restricted to females [29]. Like cardiomyocytes or neurons, podocytes have limited regenerative capacity. A deeper understanding of cell-type specific mechanisms and sex-specific responses to rapamycin might have important clinical consequences in the era of personalized medicine. Furthermore, disturbances in mTOR signaling occur in many kidney pathologies [17, 20, 30]. By using a pharmacological challenge targeting mTOR pathway with rapamycin we aimed to elucidate both, potential pathologically and physiologically relevant sex-specific responses. Using unbiased omics approaches, we provide detailed genomic data by deep sequencing of male and female podocytes under homeostatic condition and in response to mTOR inhibition. These studies were complemented with podocyte proteomics and metabolomics. The data reveal a so far unknown number of sexually dimorphic podocyte genes reported to be involved in the pathogenesis of kidney diseases and novel aspects of sexually dimorphic genomic and metabolic responses towards mTOR inhibition. These comprehensive data provide novel targets and rationale for future specific disease model studies in the field of sexually dimorphic glomerular disease manifestation and progression.

## Methods

### Mice

Gt(ROSA)26Sortm4(ACTB-tdTomato,-EGFP)Luo/J mice were purchased from The Jackson Laboratory (Bar Harbour, Massachusetts, USA) and crossed with Tg(NPHS2-cre)295Lbh mice were bred and genotyped as previously reported [31]. Mice were housed in an SPF facility with free access to chow and water and a 12-h day/night cycle. All animal experiments were conducted according to standards and procedures approved by the local Animal Care and Use Committee (LaGeSo Berlin G0241/2015). Furthermore, the ARRIVE reporting guidelines were used [32].

### Experimental model

A total of 76 ROSAmT/mG-NPHS2 Cre mice (38 male and 38 female) aged 12–18 weeks were treated with either rapamycin (LC Laboratories, Woburn, Massachusetts; 1.5 mg rapamycin/kg BW administered intraperitoneally every third day) or vehicle over a period of 3 weeks. Urine, serum and cortex kidney tissue were harvested at day 21 of the treatment period.

Podocytes were isolated from 40 mice (male and female, 9 of each sex treated with rapamycin and 11 of each sex with vehicle) according to Boerries et al., [31] with slight modifications). Kidney cortex was digested and podocytes were isolated using fluorescence-activated cell sorting (FACS). For further details of the podocyte isolation procedure, see the Supplementary Methods.

The isolated podocytes from these 40 mice were allocated to RNA sequencing (podocytes from 22 mice), qPCR for validation of RNA sequencing (podocytes from 12 mice) and proteomics (podocytes from 6 mice). Podocytes from different mice were not pooled for those methods so that each isolated podocyte sample from one individual mouse represented one biological replicate. Due to restricted amount of podocyte material from one mouse each biological replicate could therefore only be used for one of those methods. The other 36 mice (from the 76 ROSAmT/mG-NPHS2 Cre mice) were used as follows: 8 mice were allocated to electron microscopy. From the remaining 28 mice, kidney cortex from one kidney was directly snap frozen in liquid nitrogen and later used for metabolomics and protein extraction, the other kidney was either perfused with paraformaldehyde and later used for immunohistochemistry or directly embedded for cryosections. Further details of mice sex and treatment for the different experiments including numbers and reasons of excluded samples if applicable are specified in the respective methods´ sections of the main manuscript and/or in the Supplementary Methods. Furthermore, numbers of biological replicates per analysis are indicated in the figure legends.

### RNA sequencing and transcriptomics analysis

For details regarding sample preparation of native podocytes for transcriptomics analysis, see the Supplementary Methods. Poly-A selected mRNA (NEB Next Ultra II Directional RNA Library Prep Kit) was used to generate cDNA libraries and deep RNA sequencing (2 × 75 bp, paired-end) was carried out on an Illumina HiSeq4000 system. 4–6 biological replicates of each experimental group were analyzed. Sequencing performance provided 36–46 million paired-end reads per sample detecting 84% of all transcripts uniquely mapping sequences aligned to only one single gene within the genome.

For further details on differential expression computations and bioinformatic algorithms and packages, as well as validation experiments using qPCR, see Supplementary Methods and Supplementary Figure S3.

### Proteomics analysis

For details regarding sample preparation of native podocytes for proteomics analysis, see the Supplementary Methods. Proteomics data acquisition was performed on a quadrupole Orbitrap hybrid mass spectrometer (QExactive Plus, ThermoFischer) coupled to an easynLC exactly as previously described [33]. Quantitative analysis was performed using MaxQuant from individual podocyte isolations of 3 male and 3 female vehicle mice. For further details regarding the bioinformatics analysis see the Supplementary Methods.

Validation of proteomics was performed by western blotting of podocyte-specific proteins. See Supplementary Figure S4.

### Western blot and histological analyses, and electron microcopy

Kidney cortex tissue was used for examination of protein expression and phosphorylation using specific antibodies and immunoblotting. In addition, studies on kidney morphology and ultrastructure were performed using standard tissue preparations or as reported previously [34]. For used antibodies and stains and further preparative details please see Supplementary Methods.

### Metabolomics analysis

Metabolomics was performed in collaboration with Kirwan lab, BIH Metabolomics Platform, Berlin Institute of Health at Charité–Universitätsmedizin Berlin, as described previously [35]. For tissue extractions, flash frozen cortex-enriched kidney tissue from vehicle- and rapamycin-treated male and female mice were used (5–7 biological replicates for each experimental group). For details regarding sample preparation, metabolomics and bioinformatic analyses, see the Supplementary Methods.

### Statistics

For quantitative data, statistical tests (Wilcoxon ranked sum test, and univariate linear regression using R packages, R version 4.0.0) and Prism (v9.0, Graphpad) were performed as indicated. In general, *P*-value < 0.05 was considered significant. For large-scale data, correction for multiple testing was performed as described in the respective omics method sections. The number of biological replicates and statistical tests used for analysis are further indicated in the figure legends.

## Results

### Deep transcriptomic data of male and female podocytes

**Fig. 1:**
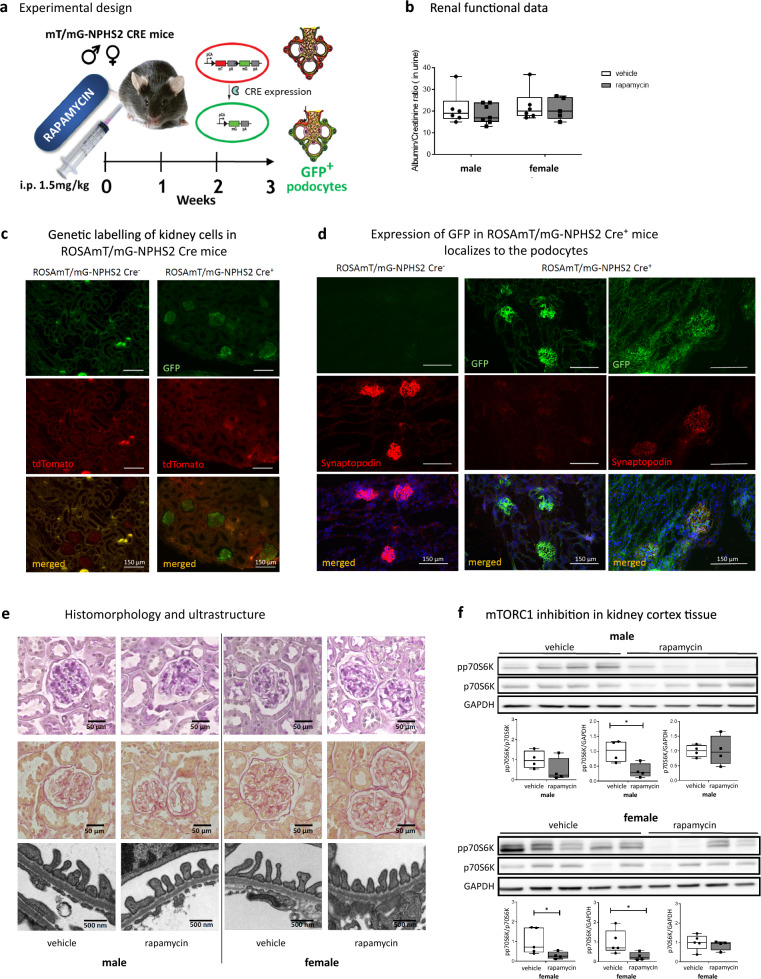
Experimental design, functional and histological model characterization. **a** Schematic representation of the experimental model. 76 male and female ROSAmT/mG-NPHS2 Cre mice were injected with rapamycin into the peritoneal cavity at a concentration of 1.5 mg/kg/BW or vehicle (DMSO) every third day during the experimental period. After three weeks, mice were sacrificed, and kidneys either flash frozen for histology and metabolomics (n = 28) or were perfused for electron microscopy (n = 8; 2 biological replicates per group) or used for podocyte isolation for qPCR and RNA sequencing (n = 34) and proteomics (n = 6). **b** Albumin/Creatinine ratio showed normal kidney function after three weeks of rapamycin in both, male and female mice (male vehicle n = 6; male rapamycin n = 11; female vehicle n = 6; female rapamycin n = 9). **c** Representative immunofluorescent images of cryo-sections of kidneys from ROSAmT/mG-NPHS2 Cre^**−**^ and ROSAmT/mG-NPHS2 Cre^**+**^ mice. Membrane-targeted GFP genetically labels NPHS2-expressing cells (podocytes) green, while all the other kidney cells are labelled red with TomatoRed (tdTomato). Scale bars, 150 µm. **d** Representative immunofluorescent images of cryosections of kidneys from ROSAmT/mG-NPHS2 Cre^**−**^ and ROSAmT/mG-NPHS2 Cre^**+**^ mice with/without indirect immunofluorescent co-staining for Synaptopodin (red). Membrane-targeted GFP genetically labels NPHS2-expressing cells (podocytes) green in Cre^+^ mice. Nuclei were stained with DAPI. Scale bars, 150 µm.​**e** Representative Periodic Acid-Schiff stainings (upper panel) and Sirius red stainings (middle panel) showing normal histomorphology with no increase in fibrosis after three weeks of rapamycin treatment in both, male and female kidney cortex tissues. Electron microscopy graphs (lower panel) displayed normal podocyte structure, foot processes, cell body, glomerular basement membrane and slit diaphragm in both sexes irrespective of treatment. Scale bars, 50 µm. **f** Representative western blots using protein extracts of podocyte-enriched kidney cortex tissues showing efficient mTORC1 inhibition in both, male and female kidneys after three weeks of rapamycin treatment as analyzed by mTORC1 downstream phosphorylation of p70S6K at Thr389 (pp70S6k) at Thr389 (n = 4–5 biological replicates per group). Bar graphs below display results of densitometric analysis with normalization of values to GAPDH and p70S6k. Mann–Whitney test was used to determine significant differences between vehicle and rapamycin treatment groups in each sex, * *P*-value < 0.05, ** *P*-value < 0.01

To characterize the sex-specific podocyte transcriptome under homeostatic conditions and in response to mTOR inhibition, male and female ROSAmT/mG-NPHS2 Cre mice were either treated with vehicle or rapamycin for three weeks and highly purified podocytes were obtained using fluorescence-activated cell sorting [31] (Fig. 1a, c, d). Trough concentrations of rapamycin were monitored and were within the range of clinically approved therapeutic levels [36] in all treated mice; 24.62 ± 3.45 and 21.6 ± 1.9 ng/ml, in male and female, respectively. Structural and functional kidney parameters are reported in Supplementary Table S1. Female mice displayed lower kidney and lower body weight compared to male mice. Rapamycin treatment induced slight non-significant body weight losses in both sexes. However, kidney/body weight ratio only significantly decreased in female rapamycin mice compared to female vehicle mice. Albumin and creatinine clearance were within the normal range in all experimental groups at the end of the experimental period (Supplementary Table S1, Fig. 1b).

Efficient mTORC1 inhibition was confirmed in kidney cortex tissues in both sexes (Fig. 1f). Interestingly, at the end of the treatment period none of the mice showed pathological alterations in kidney function, kidney cortex structure and podocyte morphology or ultrastructure (Supplementary Table S1, Fig. 1e).

**Fig. 2:**
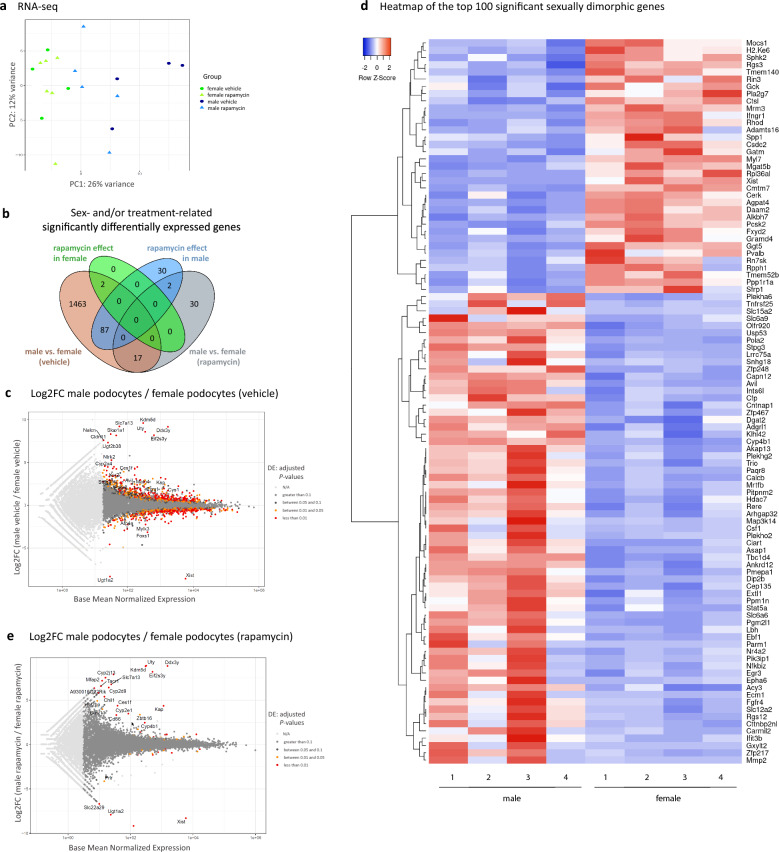
Characterization of the intrinsic sexually dimorphic podocyte transcriptome. **a** Principal component analysis of the top 500 most varying genes revealed sex-dependent separation of transcriptomes between vehicle mice (blue color indicates male podocytes, green color female podocytes; vehicle groups are represented by circles, rapamycin-treated by triangles), n = 4 male and female vehicle each; n = 5 male rapamycin treated and n = 6 female rapamycin treated). **b** Venn diagram demonstrating common and unique significantly sex-differently expressed genes between indicated comparisons (brown: male vs. female vehicle-treated group, grey: male vs. female rapamycin-treated group, green: female rapamycin vs. female vehicle, blue: male rapamycin vs. male vehicle). **c** Scatter plot showing distribution of gene copy numbers of podocytes from vehicle groups as base mean normalized expression over all samples versus log2FC male vehicle/female vehicle. Colors indicate significance levels (red: adjusted *P*-value < 0.01, orange: adjusted* P*-value between 0.01 and 0.05, dark grey: adjusted *P*-value between 0.05 and 0.1, medium/light grey: non-significantly differently expressed). **d** Heatmap of the top 100 of intrinsically sexually dimorphic genes in male versus female podocytes (adjusted *P*-value < 0.01 and expressed with TPM > 1 in all biological replicates) The four Y-chromosomal genes (*Kdm5d*, *Eif2s3y*, *Uty* and *Ddx3y*) are not shown in this heatmap. Blue indicates decreased expression level, white no regulation, red increased gene expression level. These genes are listed with respective Log2FC and TPMs in the Supplementary Table S3, sheet Top100_DE_p < 0.01_TPM > 1. **e** Scatter plot showing distribution of gene copy numbers of podocytes from rapamycin groups as normalized counts per transcript versus log2FC male/female. Colors indicate significance levels (red: adjusted *P*-value < 0.01, orange: adjusted* P*-value between 0.01 and 0.05, dark grey: adjusted *P*-value between 0.05 and 0.1, medium/light grey: non-significantly differently expressed)

In contrast to the unchanged physiological and structural findings, RNA sequencing revealed a significant separation of male and female transcriptomes under vehicle homeostatic conditions and remarkable sex-specific response patterns towards rapamycin treatment. Transcriptional responses to mTOR inhibition surprisingly abolished the clear separation by sex as demonstrated in principal component 1 (PC1) of principal component analysis (PCA) (Fig. 2a).

Overall, 1,569 genes were intrinsically significantly differentially expressed (DE) in male and female podocytes (adjusted *P*-value < 0.05) (Fig. 2b, c Supplementary Table S2), 886 genes were significantly higher expressed in female, 683 in male.

A heatmap of the top 100 of intrinsically sexually dimorphic genes (adjusted *P*-value < 0.01, expressed with Transcript per Million (TPM) > 1 in all biological replicates, therefore without the four only in male expressed Y-chromosomal genes *Kdm5d*, *Eif2s3y*, *Uty* and *Ddx3y*) is shown in Fig. 2d. Genes presented in the heatmap are listed in Supplementary Table S3, sheet Top100_DE_veh_p < 0.01_TPM > 1.

Concordant to visualization of loss of clear sex differences after rapamycin treatment in PCA (Fig. 2a), MA plot of distribution of sex-significantly DE genes in male and female rapamycin treated podocytes revealed only few sexually dimorphic genes, including mainly sex chromosomal genes (Fig. 2e).

To exclude contaminations with different cell types of the glomeruli during the purification process as a cause for the strong intrinsic sexual dimorphism, we compared our podocyte transcriptome with available kidney single cell data [37] (Supplementary Figure S2b). Furthermore, we matched our sequencing results to different podocyte specific RNAseq datasets [31, 38–40] and found high conformity between podocyte-specific transcripts further proving validity of the method (Supplementary Figure S2c). Additional experimental validation of transcriptomic data was performed by qPCR using independent cell isolations (Supplementary Figure S3).

Sexual dimorphism has been reported to be largely determined by the sex chromosomes genotype of the organism or sex hormonal regulations [41–43]. However, we could not relate the strong sexually dimorphic genes to specific positions on the sex chromosomes (apart from four genes uniquely expressed on male Y-chromosome). Interesting sub-clustering was observed, probably due to the mitochondrial expression which approximately split vehicle from rapamycin samples of each sex (Supplementary Figure S2d).

To address possible sex hormonal regulations, we analyzed our intrinsically sexually dimorphic genes for known estrogen target genes [44, 45]. We found that out of more than 500 annotated genes corresponding to estrogen target genes pathways, 54 were intrinsically sexually dimorphic (37 up in male/17 up in female vehicle podocytes) (Supplementary Table S4), whereas the vast majority of these reported estrogen target genes were not significantly sex-differently expressed. Furthermore, only 6 estrogen target genes (*Daam2*, *Egf*, *Sgk3*, *Ago1*, *Il1r1*, *Tgfa*) were significantly differentially regulated by rapamycin treatment in males and only one in females (*Ankrd33b*). This indicates that a large part of our observed sex-differences and sexually dimorphic responses to rapamycin in podocytes could not be attributed to direct genomic estrogen effects. Possible mechanism for sex-biased gene expression might also occur through sex-biased transcription factors (TFs) [43, 46]. We could identify 83 intrinsically sexually dimorphic transcriptional regulators (Supplementary Table S5) which are important candidates for regulation of sexual dimorphic gene expression.

### Functional characterization of the intrinsically sexually dimorphic podocyte transcriptome

**Fig. 3:**
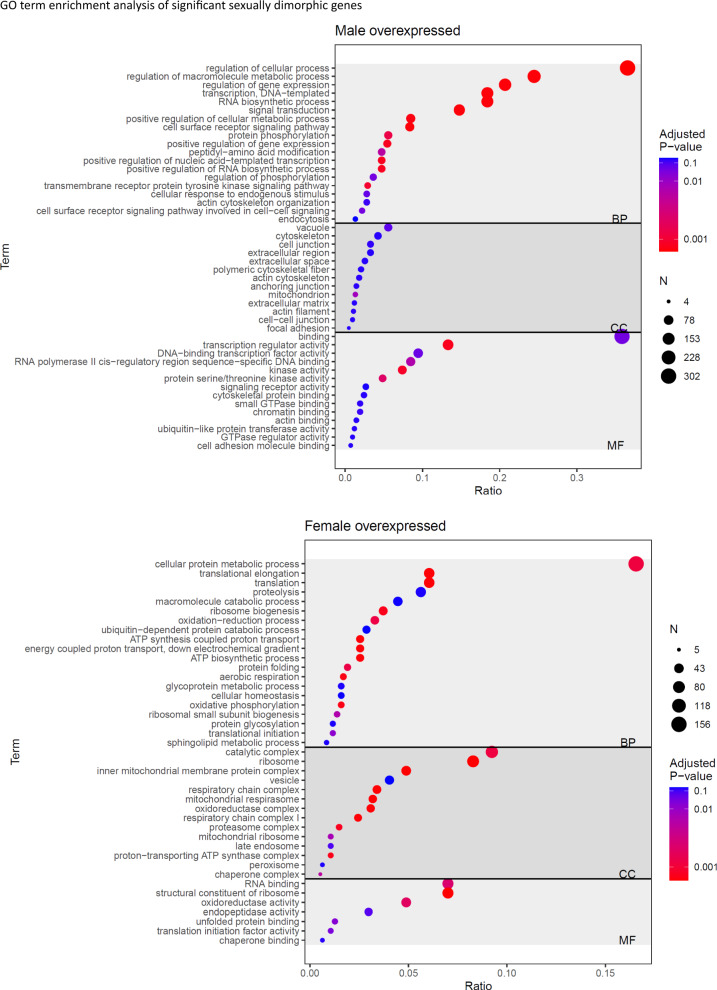
Functional characterization of significant sexually dimorphic genes. GO-slim Panther term enrichment analysis of the significantly overexpressed gene ontology terms of the 1,569 sex-DE genes for biological Process (upper in male, lower in female). (BP), molecular function (MF) and cellular component (CC). The size of the circles indicates the number of the significantly differently expressed genes in each GO (adjusted *P*-value < 0.05, FDR < 0.1)

Panther Protein Class categorization revealed that the GOs “Transporters”, “cytoskeletal protein”, “protein modifying enzymes/binding activity and transcriptional regulator" were the main significantly sexual dimorphic GOs. Further functional annotation of the DE genes (*P*-value < 0.05, ≥ 4 genes detected by panther gene ontology (GO) slim) revealed that distinct GOs were enriched in either female or male podocytes. Major gene sets overexpressed in females were related to “oxidation phosphorylation”, “translation” and “ribosome”, whereas in males “gene expression”, “kinase activity”, “cytoskeleton”, “actin cytoskeleton organization” and “cell junction” were enriched (Fig. 3, Supplementary Table S6).

### Sexually dimorphic genes related to kidney diseases

**Fig. 4:**
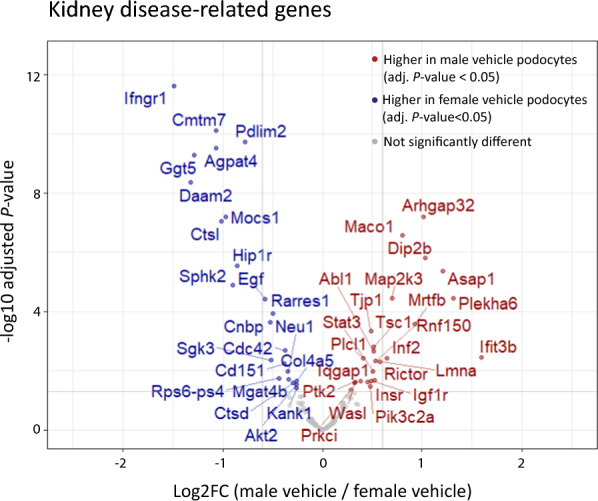
Kidney disease-related genes. Volcano plot displaying known kidney disease-related genes with gene names indicated for intrinsically sexually dimorphic genes (adjusted *P*-value < 0.05, TPM > 1 in all vehicle podocytes): the y-axis shows the statistical significance measured by –log10 adjusted *P*-value, and the x-axis shows the log2FC of male versus female vehicle podocytes (> 1 reflects higher than 2 FC in male podocytes; -1 means twofold lower in male podocytes). Each red point represents an individual gene that is intrinsically significantly higher expressed in male podocytes; each blue circle represents a specific gene intrinsically significantly lower expressed in male podocytes. (male vehicle n = 4, female vehicle n = 4, male rapamycin n = 5, female rapamycin n = 6)

Structural and functional integrity of the podocyte is strongly dependent on proper organization of its cytoskeleton and fulfillment of metabolic requirements, GO terms which appeared to be enriched in our sex DE analyses. To investigate clinical impact of these sexually dimorphic genes, we extended our analyses of intrinsically DE genes in podocytes to genes reported to be involved in the pathogenesis of kidney diseases. We used published data to determine any gene overlaps [47–58]. Out of 191 genes with described roles in kidney diseases, 161 were expressed with an abundance of at least 1 TPM in all vehicle podocytes, among them 47 genes with intrinsic significant sexual dimorphic expression (adjusted *P*-value < 0.05) (Fig. 4, Supplementary Table S7). Interestingly, only six out of these 47 intrinsically sexually dimorphic reported disease genes could be identified as estrogen target genes (Supplementary Table S4). A heatmap of the expression levels of these estrogen target genes (*Akt2, Ctsd, Egf, Igf1r, Kank1 and Daam2)*, as well as for other functional groups of intrinsically sex DE disease genes related to kidney disease are shown in Supplementary Figure S5. Respective genes are separately presented for transcriptional regulators, cytoskeleton-related genes, metabolism & protein turnover and signaling and kinases.

### Sex-specific transcriptional changes induced by mTOR inhibition

**Fig. 5:**
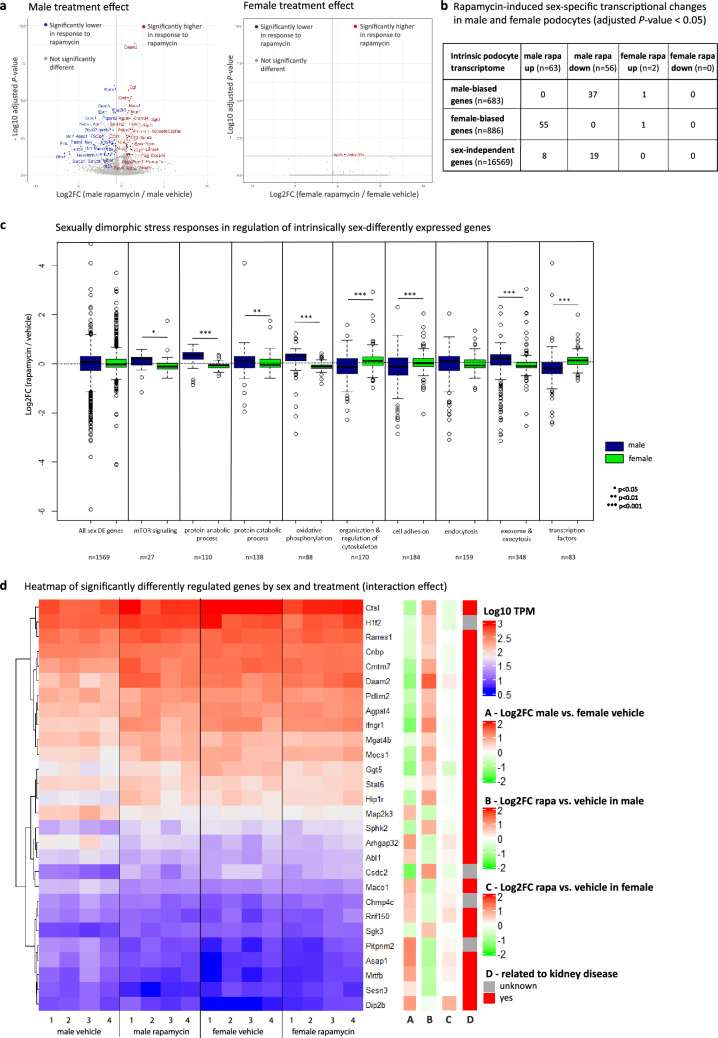
Sex-specific transcriptional changes induced by mTOR inhibition. **a** Volcano plots displaying rapamycin treatment effects in each sex separately (left graph representing results in male podocytes, right graph in female podocytes): the y-axis shows the statistical significance measured by –log10 adjusted *P*-value, and the x-axis shows the log2FC of rapamycin versus vehicle treated male, respectively rapamycin versus vehicle treated female podocytes (> 1 reflects higher 2 FC in rapamycin treated podocytes; -1 means twofold lower in rapamycin-treated podocytes). Each red point represents an individual gene that is significantly higher expressed in rapamycin-treated podocytes (adjusted *P*-value < 0.05); each blue circle represents a specific gene significantly lower expressed in rapamycin-treated podocytes. (male vehicle n = 4, female vehicle n = 4, male rapamycin n = 5, female rapamycin n = 6). **b** Table of by sex-bias grouped gene numbers significantly changed by rapamycin in each sex. **c** Graphs of sex-specific treatment changes of intrinsically sexually dimorphic genes for selected enriched GO terms. n = number of genes within specific term, significance levels * *P*-value < 0.05, ** *P*-value < 0.01, *** *P*-value < 0.001 as determined by Wilcoxon ranked sum test. (male vehicle n = 4, female vehicle n = 4, male rapamycin n = 5, female rapamycin n = 6). **d** Heatmap of the expression level and log2FC of significantly differently regulated genes by sex and treatment (interaction effect). Blue indicates decreased expression level, white no regulation, red increased gene expression level. (Each 4 biological replicates per group)

We first analyzed the effects of rapamycin treatment in each sex separately. Remarkably, male podocytes showed after exclusion of rhythmic genes DE of 119 genes (63 up/56 down, *P*-value < 0.05) and females only 2 DE genes (Fig. 5a). These data suggest that female podocytes may maintain a more stable transcriptome under the challenge of mTOR inhibition with rapamycin in contrast to male podocytes which underwent much more drastic shifts in expression level changes. Furthermore interestingly, 87% of genes significantly upregulated by rapamycin in males belonged to the female-biased genes and 66% of the significantly downregulated genes in males were male-biased genes (Fig. 5b). This suggests that interference with mTOR signaling affected intrinsically sex-biased gene expression to a greater extent than sex-independent gene expression.

Separate male and female subgroup analysis was undertaken of the intrinsically sex DE genes after rapamycin treatment. This revealed that the most prominent changes after treatment involved the intrinsically significantly different genes such as the sex different stress responses in genes related to “mTOR signaling”, “protein anabolic and catabolic process”, “oxidative phosphorylation”, “organization and regulation of cytoskeleton”, “cell adhesion”, “exosome and exocytosis” and “transcription factors” (Fig. 5c).

Further Gene set enrichment analysis (GSEA) of the complete male and female transcriptomes revealed male-biased downregulation of gene sets related to many signaling pathways, inflammation and metabolism in response to rapamycin (Supplementary Table S8). In contrast, gene sets related to amino acid transport and metabolism appeared downregulated in both male and female podocytes, yet to a greater extent in males. We extended our analyses of effects of mTOR inhibition to canonical pathways´ activations in male and female podocytes with the use of QIAGEN IPA (QIAGEN Inc., https://digitalinsights.qiagen.com/IPA) [59]. Interestingly, many of the pathways showed significant changes in only one sex or in opposite directions, such as gene sets related to TCA cycle, Granzyme A, PDGF, Paxillin and RAAS and Sirtuin signaling pathways (Supplementary Table S9).

To get functional insight into these sex-different pathway and gene set activations induced by mTOR inhibition, further upstream regulator analyses were performed. With a Z-score of <|2| and *P*-value of overlap < 0.05, a total of 115 and 55 potential upstream regulators were identified in male and female, respectively (Supplementary Table S9). Among them were several genes known to be involved in kidney diseases, such as *Kdm5a*, *Rb1*, *Tead1, Xbp1* and *Ppargc1α* with so far unknown role as sexual dimorphism regulators [60–69].

Interaction effect analysis considering sex and treatment resulted in only 28 DE genes, most of them only significantly altered in males (Fig. 5d, Supplementary Table S2). Remarkably, 86% have been previously reported in relation to kidney pathologies and many of them are involved in cytoskeleton remodeling or represent transcriptional regulators.

Considering the fact that mTOR has important effects on immunity [70–72], we additionally focused on inflammation and immune aspects of the podocytes. We did not induce any inflammatory condition or immune disease in our model, thereby we did neither expect nor found high gene expression levels of cytokines and immune-related receptors in both, control and rapamycin -treated mice. Along this line, we could not detect any lymphocyte infiltration in kidney cortex tissue in histological stainings of any of the four experimental groups. Nevertheless, in order to focus further on aspects of the “immune podocyte”, we merged our gene expression lists with genes related to immunity in podocytes as reported in Bruno et al. [73] (see sex- and treatment related DE sublists of these genes presented in Supplementary Table S10). Under these genes, we only found complement 3 (*C3*) to be significantly sexually dimorphic with higher expression in male podocytes compared to female podocytes and a trend towards significant downregulation of *C3* in male in response to rapamycin treatment. Further, GSEA analysis showed that several GOs related to inflammation and immune response were regulated by rapamycin in both sexes, especially many of them significantly downregulated in male after rapamycin (see Supplementary Table 8, sheet “GOs related inflammatory and immune response”).

### Characterization of the sexually dimorphic intrinsic podocyte proteome

**Fig. 6:**
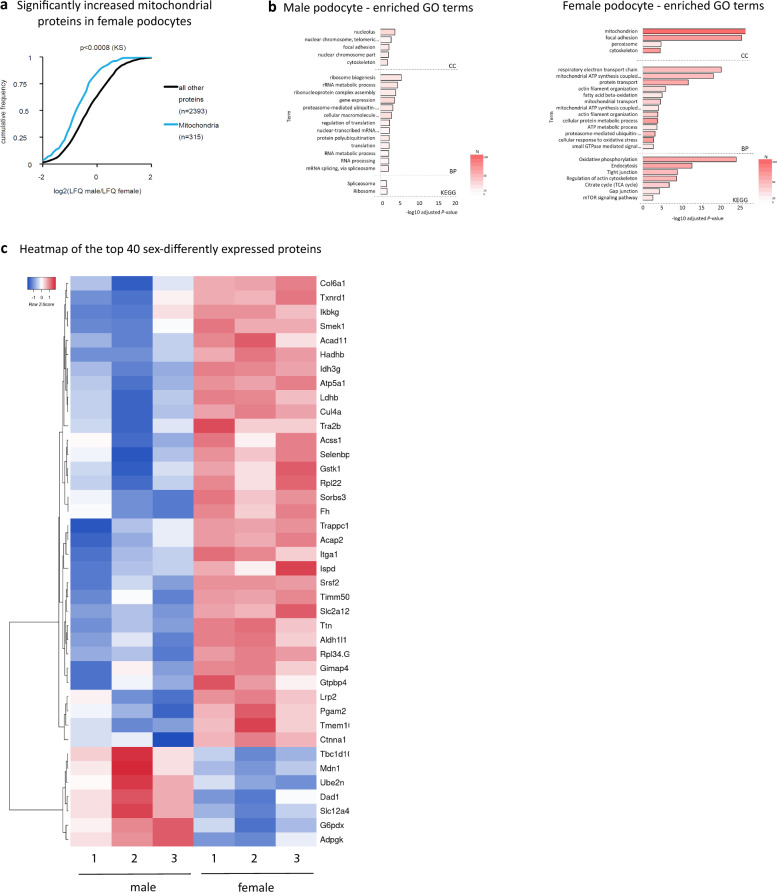
Characterization of the sexually dimorphic intrinsic podocyte proteome. **a** Podocyte-specific proteomics. Female podocytes showed significantly higher expression of total mitochondrial proteins compared to male podocytes (n = 3 biological replicates per each vehicle group). **b** EnrichR analysis of proteins with sex-differential expression (FC > 2) showed major hits in terms related to transcription and protein translation/turnover in male podocytes, whereas females had concordantly to the female transcriptome enrichment in mitochondrial proteins and increased cytoskeletal and cytoskeleton-regulating proteins. Left panel shows enriched terms for cellular component (CC), biological process (BP) and KEGG pathway (KEGG) in male podocytes, right panel respective enriched terms in female podocytes, adjusted *P*-value < 0.05, N = number of proteins within specific term. **c** Heatmap of the top 40 of intrinsically sexually dimorphic proteins in male versus female podocytes (adjusted *P*-value < 0.05) showed that a higher number of genes were enriched in female podocytes. Blue indicates decreased expression level, white no regulation, red increased gene expression level (n = 3 biological replicates per vehicle group)

Interestingly, proteomics analysis of isolated vehicle-treated male and female podocyte proteins confirmed significant enrichment of mitochondrial proteins in female podocytes (Fig. 6a, Supplementary Table S11). Proteins with sex-differential expression (FC > 2) were further analyzed using EnrichR. Concordant with the transcriptome, females had, in addition to enrichment in mitochondrial proteins, increased cytoskeletal and cytoskeleton-regulating proteins (Supplementary Fig. 4), whereas major hits in male podocytes were related to transcription and proteostasis (Fig. 6b). The higher number of cytoskeletal and cytoskeleton-regulating proteins in female compared to male podocytes suggest a higher biophysical resilience to adapt to mechanical forces in their environment and therefore better maintenance of glomerular function in females under stress conditions [49, 74, 75].

Concordant with transcriptomics, GO terms “oxidative phosphorylation”, “Glycolysis”, “proteasome” and “mitochondria” were overrepresented in the dataset from female podocytes. Yet, as expected for comparisons of complex samples [76], the complete proteomes of male and female podocytes did not correlate strongly with their respective transcriptomes (Spearman rank coefficient in females: 0.43, in males: 0.39, p-values < 2.2e−16). Interestingly, the subsets of sexually dimorphic genes related to mitochondria/oxidative phosphorylation, correlated moderately with protein expressions in both, male and female podocytes (Spearman rank coefficient in females: 0.52, in males: 0.51, p-values < 2.1e−08). A heatmap of the top 40 significant sex-differently expressed proteins is presented in Fig. 6c.

### Sex differences in energy metabolism

**Fig. 7:**
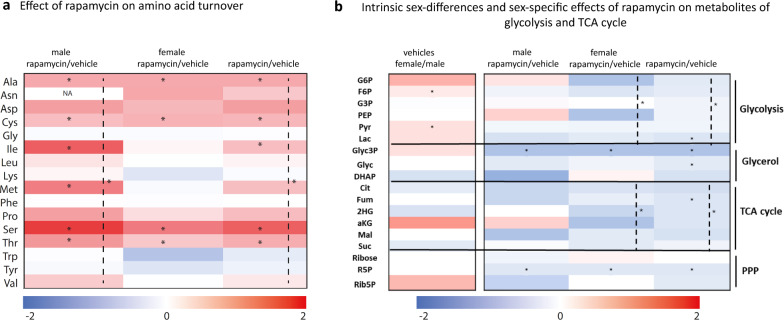
Sex differences in energy metabolism. **a** Heatmap of log2FC of the mean of the normalized peak areas of amino acids in male and female kidney cortex tissue in response to rapamycin in each sex separately and male and female combined (rapamycin/vehicle). Blue indicates decreased levels, white no regulation, red increased accumulation of metabolites (n = 6 male vehicle; n = 6 male rapamycin treated; n = 7 female vehicle and n = 5 female rapamycin treated n = 6), * *P*-value < 0.05. Dashed line represents significance for amino acids after univariate scaling. **b** Heatmap of log2FC of the mean of the normalized peak areas of glycolysis metabolites, TCA cycle, pentose phosphate pathways and others in male and female kidney cortex tissue in male and female vehicle, response to rapamycin in each sex separately and male and female combined (rapamycin/vehicle). Blue indicates decreased levels, white no regulation, red increased accumulation of metabolites. n = 6 biological replicates in each group, * P-value < 0.05. Dashed line represents significance for the group of glycolysis and TCA after univariate scaling

GSEA of rapamycin effects on the whole male and female transcriptome had revealed that TCA cycle, carbohydrate derivative biosynthesis process and oxidation phosphorylation as well as several other metabolic processes decreased in females whereas males mostly responded conversely (Supplementary Table S8) suggesting sex-different stress responses. This, together with the high number of sexually dimorphic mitochondrial and translational genes and proteins prompted us to further study metabolic consequences of mTOR inhibition in male and female mice. Metabolomics was performed directly from podocyte enriched snap frozen kidney cortex tissue to ensure valid results of the in vivo changes induced by rapamycin. Data were analyzed to confirm correct metabolite annotation and for outliers (Supplementary Methods). Sixteen statistically significant (p < 0.05) outliers were removed. As expected, concordant with the well-known negative effect of mTOR inhibition on protein synthesis[77], significant accumulation of most amino acids occurred in both sexes, yet to a higher extent in males (*P*-value < 0.05) (Fig. 7a). In addition, metabolomics pointed to intrinsically increased glycolytic metabolites in female compared to male. Rapamycin reduced glycolysis and TCA cycle in both male and female. However, this reduction in response to rapamycin was only significant in females (Fig. 7b, Supplementary Figure S6). Phosphorylation of AMPK as indicator for the general energy status revealed a tendency towards higher levels in female compared to male, yet due to high interindividual variability especially in the female group this difference did not reach statistical significance (p-value = 0.07, Supplementary Figure S7). Both sexes maintained pAMPK levels in response to rapamycin, yet male showed a tendence towards higher AMPK phosphorylation after rapamycin treatment. Total AMPK did not show intrinsic sex-differences and did not change by rapamycin treatment in both, male and female. (Supplementary Figure S7).

## Discussion

Podocytes are an essential part of the filtration barrier and any pathological alteration likely leads to proteinuria and serious pathologies [1, 74, 78]. Despite of high sexual dimorphism in many glomerular kidney disease prevalence and progression [2–4, 6], sex-specific molecular identity of podocytes remained so far unknown. A recent paper in Nature Methods called attention to that frequent limitation of molecular omics studies and requested sex annotation for fully accounting for the biology of sex differences [11]. Our study aimed at closing this knowledge gap in podocyte biology. We addressed intrinsic gene transcription and protein expression in murine male and female podocytes and determined sex-differences in podocyte responses towards pharmacologic intervention with rapamycin. Remarkably, many genes previously reported in podocytopathies showed so far unknown sexual dimorphic expression and transcriptional changes after rapamycin which might account for sexual dimorphic disease susceptibilities.

Our data suggest that female podocytes may have a more stable transcriptome considering the challenge of mTOR inhibition whereas males induce earlier molecular and metabolic changes. The mitochondrial energy providing machinery, endocytosis and exocytosis, and cytoskeletal integrity were major targets of transcriptional changes that were more strikingly affected in male podocytes. Interestingly, mTOR inhibition largely abrogated the clear separation of intrinsically sexually dimorphic genes in podocytes, mainly by changes in the male transcriptome.

Our stress model did not induce structural or kidney functional changes suggesting that the observed differences in podocytes might reflect early endogenous sex-specific compensatory mechanisms prior to disease development. The lack of functional changes contrasts with other reports where rapamycin induces proteinuria [20, 79]. This difference might be explained by the different mouse strains used in these studies. Other studies using C57BL/6 mice report lack of development of proteinuria by mTORC1 inhibition similar to our work [80]. Furthermore, a recent report shows that rapamycin might also delay development of proteinuria in specific disease models [81] suggesting that rapamycin effect on kidney functional changes is highly complex, mouse-strain- and context-dependent.

Female mice displayed lower kidney and lower body weight compared to male mice, which is considered physiological in these strains and moreover applies also to human [82–85]. Furthermore, rapamycin has already been shown to induce significant reductions in kidney/body weight ratio in several disease models [86–88] interestingly in some studies, similar to our study, to a higher degree in female compared to male mice [83]. Weight loss in response to rapamycin treatment has been attributed to central effects on mTORC1 inhibition in the hypothalamus which leads to decreased food intake [89, 90]. In addition, rapamycin regulates energy homeostasis by leading to enhanced insulin sensitivity [91]which causes less blood sugar increases, thereby reducing hunger and craving for food. Another reason for reduced food intake and subsequent weight loss under rapamycin treatment may be the regulation of gastric ghrelin production [92]. Unfortunately, we did not control for food intake in our mice that we were unable to report differences due to calorie input as possible cause for the reduction in body weight under rapamycin treatment. However, the weight losses were within the range of those reported previously in various mouse models and we did not observe any rapid weight losses that we do not consider that as a sign of rapamycin toxicity or over-inhibition of mTOR. The cause for the unproportional greater kidney weight loss compared to body weight loss in rapamycin-treated females remains elusive. One might speculate an impact of sex hormones [93, 94]. mTOR inhibition affected estrogen receptors and androgen receptor gene expression to different extents in our study which might have contributed directly or indirectly to these sex-differences in kidney weight loss.

Although major sex differences in gene expression have been generally attributed to sex chromosomes and sex-hormonal effects [42] most of the intrinsic sex differences in podocytes observed in this study could not be associated with their position on sex chromosomes or direct estrogen target effects. Previously, it has been reported in other species that autosomes house the majority of genes with sex-biased expression [95]. Furthermore, the male-predominant idiopathic nephrotic syndrome in children of 0–18 years of age supports the fact that factors other than hormonal effects contribute to at least some sexual dimorphic podocytopathies [50].

Recently, it has been reported that beyond chromosomes and sex-hormonal effects, several TF show directional agreement of the target genes between the activating or repressive effect of the TF, the sex bias of the TF, and the sex bias of the target gene and may account for about 27% of lineage-specific sex bias [42, 96, 97]. In our study, we could identify 83 transcriptional regulators of intrinsically sexually dimorphic genes in podocytes, among them *Stat5a*, which has been recently demonstrated to regulate 23% of female-predominant genes in mouse liver [96]. Thus, regulation by sex-biased transcription factors most likely contributed essentially to our observed sex differences in podocytes.

Our differential expression analysis taking sex and treatment differences into account revealed further TF of interest, such as *Mrtfb, Cnbp* and *Stat6,* which appeared upregulated in males and downregulated in female podocytes in response to rapamycin. *Stat6* has previously been reported as regulator of cyst growth in polycystic kidney disease [98] and implicated in kidney fibrosis in a model of unilateral ureteral obstruction [99], yet further data on sex differences and functional role in podocytes are still lacking.

Other transcription factor candidates for sexual dimorphic gene expression patterns in our study identified by IPA were e.g. *Xbp1*, and *KDM5a*. *Kdm5a* mediates kidney failure in lipopolysaccharide-induced sepsis of mice [100], induces stem-like cancer cells and promotes renal cell carcinoma [62, 101]. Via activating or repressing transcription in demethylase-dependent or independent manners [101], it acts in both, homeostasis and disease [60, 61]. Kdm5a inhibitors are already tested in clinical trials [102]. Thereby, some of our observed intrinsic sex differences might have been mediated by epigenetic events [16, 41, 103] and might be an interesting future therapeutic target.

Recent evidence points to sex differences in circadian endocrine rhythms and oscillation of clock genes [104–106], which might be relevant for sexual dimorphic features of podocytopathies. E.g. *Arntl* has been shown to be critical for genes involved in podocyte integrity, metabolism and adhesion [107] and the transcription factor *Creb3l1* has recently been demonstrated in male podocyte injury and degeneration [108]. However, our study was not specifically designed to investigate such differences. Taking into consideration that sex differences do not only appear in expression levels at specific time points during the day but also might significantly peak earlier in one sex [106], future studies require investigating multiple different day times in addition to well-controlled times for sacrifice of mice.

Remarkably, many of the intrinsically sexually dimorphic and significantly deregulated genes after rapamycin treatment in this study were kidney disease-related. Yet, to our knowledge, none of the genes that were significantly differentially regulated considering sex and treatment have been investigated for sexual dimorphism in kidney cells so far. Interestingly, some of them were reported to be sexually dimorphic in other tissues, such as in microglia and skeletal muscle [76, 109]. Among them, *Daam2, Pdlim2*, *Asap1*, and *Sphk2* all of which have a known relationship to kidney diseases [110–114]. *Daam2*, a gene involved in actin remodeling with certain variants causing nephrotic syndrome [111], as well as *Cd151*, important for podocyte-basement membrane attachment [115–117] were intrinsically significantly higher expressed in female podocytes and significantly increased only in male podocytes after rapamycin treatment. Interestingly, *Daam2* and *Cd151* have recently been reported to be upregulated in a dataset of human focal glomerulosclerosis [118]. Beyond other genes in our study, sexually dimorphic response patterns were observed for *PGC-1α*, a master regulator of mitochondrial biogenesis and transcriptional factor relevant for podocyte homeostasis [119] and a known role in kidney diseases [65], and *Ctsl,* encoding a cysteine protease which promotes Cd2ap and synaptopodin proteolysis [57, 120]. It has been reported that podocyte pH modulation by glutamine supplementation reduces cytosolic cathepsin L protease activity and can reduce foot process effacement and proteinuria [121]. We did not measure pH in our isolated podocytes and were not able to determine cathepsin L levels in our podocyte-specific proteomics of vehicle groups. Furthermore, due to the possibility of conversion of glutamate during the autosampler and derivatization during LC–MS/MS measurements [122], trustable glutamine and glutamic acid levels could not be reported in our metabolomics study and we were therefore unable to make any statement about glutamine level in male and female vehicle and rapamycin groups. To approach this important question for functional relevance of rapamycin effects on cathepsin L further, future studies are warranted including pH and enzyme activity measurements into their study protocols.

Another important protein involved in actin-regulation of podocyte foot processes is Pdlim2 [113]. It has been recently validated as a podocyte-specific protein in stainings from the Human Protein Atlas [118]. By correlation of a set of podocyte-specific genes with GFR in a patient cohort with glomerular diseases the potential of these genes to be used as candidate markers for disease progression has been demonstrated [118]. A further gene of interest among the top 100 intrinsically sex-differently expressed podocyte genes that showed sex-different responses to rapamycin is *Sphk2*. Sphk2 has been shown to be involved in kidney fibrosis and diabetes-induced podocytopathy and has recently been suggested as pharmaceutical target to treat proteinuric kidney disease [123, 124].

Considering the sexual dimorphic expression of many of the genes identified in our study strengthens the need that further candidate marker studies should include sex as a biological variable in their design.

The significant transcriptional changes in male podocytes suggest a stronger dependence on intact mTOR signaling in males to maintain podocyte homeostasis. This was also observed at the metabolomic level, where genes involved in amino acid metabolism and transport were more suppressed in males. Furthermore, higher amino acid accumulation in male metabolomics pointed to higher reduction in protein synthesis in males compared to females. Sexual dimorphic responses to mTOR inhibition by rapamycin have also been reported on organismal level. Female mice, even when treated late in life, had a longer lifespan compared to male mice [125]. For this reason, our finding of sexual dimorphism in alpha-ketoglutarate levels is of interest. Alpha-ketoglutarate has been recently attributed a crucial role in delayed aging and improved longevity [126, 127].

Despite the fact that most studies about the effects on longevity haven´t been investigated in human, many people take rapamycin off-label [28]. Recently, Kaeberlein et al. performed an observational study comparing rapamycin users to non-rapamycin users for perceived health benefits and adverse events [128]. Mouth ulceration was the only self-reported significantly different adverse event in rapamycin users. In contrast, rapamycin users reported less abdominal cramps and pain, depression, muscle tightness, anxiety and eye pain. However, these domains except for mouth ulceration lost their significant difference when only female survey responders were included in the analysis. Although the study design was not double-blinded, placebo-controlled and no clinical laboratory results were evaluated it remains intriguing with regard to our findings to hypothesize that rapamycin effects display sexual dimorphic aspects in healthy and diseased people. Along this line, the results from an ongoing cross-sectional trial with rapamycin users versus non-users including clinical laboratory results, as well as molecular and metabolic signatures [28, 129] will be of specific interest for the translational value of our data. Yet, studies focusing on rapamycin effects in human kidneys are still scarce [25].

Concerning the treatment duration with mTOR inhibitors required to elicit functional effects, recent studies have shown rapamycin effects on longevity/aging already after a brief exposure to rapamycin of three months which was as effective as long-term treatment, despite the return of TORC1 levels to normal within 2 days of stopping rapamycin [27, 130]. These studies suggest that changes on downstream signaling induced by inhibition of mTORC1 such as induction of autophagy contribute to long lasting organismal changes and do not require persistent mTORC1 inhibition. Maintenance of autophagy or respective induction in kidney disease models with decreased activity has been shown to be cytoprotective for podocytes [131]. Therefore, time-restricted mTORC1 inhibition regimen might finally be clinically more beneficial compared to longtime treatments with mTORC1 inhibitors which still have high systemic and substantial side effects. Yet so far, we are not aware of any brief time-restricted intervention studies with rapamycin in the context of kidney diseases. Therefore translating our results to possible effects in kidney diseases we can only speculate that for the induction of a „rapamycin memory “ 2 weeks of rapamycin treatment might be too short [132], whereas any time between the 3 month treatment period for the induction of longevity effects and the 25 weeks of treatment with relevant functional and structural effects [133] might be considered for conception of such proof of concept studies for the effectiveness of time-restricted administration of rapamycin in kidney disease models. Of note, dual mTORC1/2 inhibitors, such as AZD2014, do not maintain immunoregulatory effects after drug withdrawal and thus differ from the longer lasting effects of rapamycin [134]. For the induction of changes by rapamycin on protein translation, already 2 weeks of treatment appear to be enough [132].

Another approach to reduce negative clinical side effects of rapamycin treatment beyond changes in treatment time is the development of more specific mTORC1 inhibitors [28]. Rapamycin has tissue-dependent effects on mTORC2 and at least part of its negative effects e.g. on glucose and lipid metabolism have been attributed to inhibition of mTORC2 [135]. Therefore, more specific mTORC1 inhibitors might have the potential to reduce signaling network disturbances and compensatory up-/downregulation of mTORC2 in various tissues, thereby reducing negative side effects of rapamycin. Recently, other approaches to more specifically inhibit mTORC1 compared to rapamycin have been suggested as novel therapeutic option in podocyte diseases, such as indirect modulation of mTORC1 by ketone bodies [136]. Remarkably, part of the renoprotective effect of SGLT2 inhibitors might also rely on their effect to increase circulating ketone body concentration [137]. Reduction of mTORC1 activity can also be achieved by reducing the content of dietary protein or specific dietary amino acids [138]. Altogether, the development of specific mTORC1 inhibitors appears promising for health maintenance and treatment of a variety of diseases with mTOR-pathway-related pathologies.

In addition to variations in treatment times and specificities of mTOR inhibitors, sex differences in treatment results with different dosages have to be considered. Recently, Bitto et al. [130] reported that high-dose rapamycin treatment induced aggressive hematopoietic cancer development in female but not in male mice whereas lower dosage comparably increased lifespan in both sexes. We induced mTORC1 inhibition in kidney cortex tissues of male and female mice applying the same dosage of rapamycin and treatment time and achieved comparable rapamycin trough levels. Nevertheless, molecular signatures in podocytes were sexually dimorphic.

We cannot rule out differential sex-specific mTORC1 inhibition in podocyte and non-podocyte kidney cortex cells by rapamycin. However, bioinformatic analysis of downstream target changes by rapamycin in each sex in of our RNASeq data pointed to significant mTORC1 inhibition in podocytes of both sexes. Beside direct effects of rapamycin on podocytes, indirect effects of rapamycin-induced changes in surrounding cells or more distal known effects of rapamycin, such as reported in gut bacteria [139], metabolism [140] and the immune system [141, 142] might have additionally shaped the sex-specific molecular signature of podocytes in response to systemic rapamycin treatment [28].

mTOR inhibitors have well-known effects on immunity [70–72]. Furthermore, immunity displays important sexual dimorphism [143–146]. Accumulating evidence suggests that podocytes are not only targets of immune responses but also play an active role in innate and adaptive immunity [1, 73]. They may aggravate immune and nonimmune glomerular injury through expression of receptors linked to pathways that induce proinflammatory molecules [147, 148]. Interestingly, we found *complement 3* to be significantly sexually dimorphic with higher gene expression in male podocytes compared to female podocytes and a trend towards significant downregulation of *C3* in male in response to rapamycin treatment. In human studies, C4 and its effector C3 were present at higher levels in cerebrospinal fluid and plasma in men compared to women. It was suggested that this sexual dimorphism contributed to women´s greater risk of systemic lupus erythematodes [149, 150]. Sexual dimorphism of genes involved in the complement system has been also reported in different immune cell types, such as macrophages [151].

Interestingly, mTOR inhibition affected estrogen receptors (*ESR1*, *ESR2*) and androgen receptor (*AR*) gene expression levels in male and female podocytes to different extents, which might constitute a further regulatory level for sex-biased gene expression. ERα, ERβ and AR are expressed in podocytes [152]. Downregulation of ERβ by rapamycin has previously been reported in other postmitotic cells, such as cardiomyocytes with sex-specific functional consequences [29]. Furthermore, it has recently been shown that female estrogen receptor knock-out mice developed glomerulosclerosis due to excessive ovarian testosterone production and secretion and extracellular signal-regulated kinase (ERK) pathway [152]. Therefore, balanced hormone receptor expression together with adequate sex hormone levels participate through genomic and non-genomic sex hormone effects, which needs to be considered when further investigating specific disease models.

Even if our metabolomics study and signal transduction investigations were performed only in kidney cortex tissue and we did not control for food intake and mitochondrial oxidative phosphorylation-related ATP production, our results on sex differences in metabolism are interesting especially in relation to recent studies performed in mice and human [9, 153, 154]. AMPK has been named the “guardian of metabolism and mitochondrial homeostasis” [155]. Under conditions of low energy, AMPK phosphorylates specific enzymes and growth control nodes to increase ATP generation and decrease ATP consumption [155].

In the kidney, AMPK activation may be protective against fibrosis, inflammation and mitochondrial dysfunction [156]. Furthermore, there is a close interaction between mTORC1 and AMPK signaling [157]. Concordant to our intrinsic sex difference in pAMPK levels, Lee et al. reported in a high-fat diet (HFD) mouse model decreased AMPK activity (indicated by increased phosphorylation) in male mice which were susceptible to kidney injury, whereas female mice did not display AMPK inactivation and were resistant to that obesity-induced HFD-induced kidney injury [158]. Considering the tendency of rapamycin-induced higher phosphorylation of AMPK in male in our study and their results in that disease model we may speculate that rapamycin might exert therapeutic effects in male mice.

Glycolysis and mitochondrial metabolism both contribute to podocyte energy supply. Cell environmental context and differentiation status impact on switches from fatty acid oxidation to glycolysis and vice versa [159–164].

On intrinsic transcriptional levels, we found overrepresentation of GO terms “oxidative phosphorylation”, glycolysis” “proteasome” and “mitochondria” in functional GSEA analyses in the dataset of female podocytes. This is concordant to many studies reporting higher gene expression of these groups in various female cells [165, 166]. We are not able to directly relate our gene expression results from podocytes to our data on metabolomics without additional data as already mentioned above. Yet, metabolomics of kidney cortex comparing male and female vehicle pointed to significantly increased glycolysis in females (Fig. 7b). Increased pyruvate concentration in female proximal tubular epithelial cells has been suggested to be protective against diabetic kidney disease, whereas in male enhanced TCA metabolism could be deleterious [9]. Furthermore, in adults with chronic kidney disease, increased plasma levels of TCA cycle metabolites positively associated with male sex, diabetes and all-cause mortality and negatively associated with estimated glomerular filtration rate (eGFR) [9]. In our model, rapamycin led to decreased glycolysis and TCA cycle in both, male and female kidney cortex, to a higher extent in females. The functional relevance of this finding requires further investigation in disease models.

## Limitations of the study

In this paper, we aimed to investigate the molecular signature of male and female podocytes and used a systemic pharmacological challenge with the mTOR inhibitor rapamycin to identify sex-specific response patterns in addition to podocyte cell intrinsically sex differences. Undoubtedly, there are also limitations associated with this approach. On one side, systemic drug administration reflects the frequent clinical situation where drugs affect not only the respective target cells but additionally other cell-types. However, by that, the molecular changes observed in podocytes might not be a direct drug effect, but also depend on the surrounding tissue or circulating factors which might themselves be sex-specific and therefore might have indirectly changed the male and female podocyte transcriptomes. Another limitation of our study is the cross-sectional design. Our work focuses on characterization of sex-differences on different molecular levels within one mouse strain at one age group with one specific rapamycin concentration for a fixed treatment time. Taking into account the fact that targeting mTOR signaling has different effects at various age groups [167–169], different age groups should be included in future studies when addressing therapeutic effects of mTOR inhibition.

In addition, isolation of primary podocytes from mouse tissues requires several preparative steps. This results in immediate early gene activation which might have further impacted on the molecular changes of the podocytes even if this stressor appeared to be comparable between all groups (Supplementary Table S12). This also prevented us from performing immunoblotting from isolated podocytes to assess phosphorylation of specific proteins in addition to the fact that isolated podocytes from one mouse do not yield enough material to apply such method. For that it is quite common in podocyte studies, that kidney cortex material is used instead [170–173]. Also, for metabolomics, it is important to directly harvest material after animal sacrifice. We therefore had to accept the limitation not to use the isolated podocytes, but instead kidney cortex tissue. On one side, this allowed us to obtain high quality material for metabolomics, but on the other side we had to accept that we thereby could not evaluate sexual dimorphism at the molecular level of the podocytes but only podocytes together with podocyte-adjacent cells in kidney cortex material. Moreover, to overcome at least partly this restriction, our studies were complemented with functional analyses of podocyte transcriptomes to indirectly assess podocyte-specific mTORC1 inhibitory effects and metabolic consequences of mTOR inhibition.

Furthermore, the fact that podocytes, even if crucially important for kidney function, belong to the low represented cells of the kidney make it difficult to compare our results with previous single cell studies in the kidney [37]. The correlation of our sexual dimorphic genes to a recently published single cell dataset from male and female mouse kidneys [174] only showed poor correlation (data not shown). Yet, podocytes were underrepresented in their study, underlining the fact that sexual dimorphism occurs cell-type specifically [118]. Interestingly, some of the intrinsically sexually dimorphic podocyte genes have been recently reported to be sex-differently expressed in cells of the renin lineage [175]. Univariate linear regression analysis revealed high correlation of the reported log2 fold change (FC) in renin lineage cells with our data (Supplementary Figure S1a).

## Perspectives and significance

Overall, this work provides a deep insight into sexual dimorphism of podocytes. Our study revealed that female podocytes show a more resistant profile to the effects of mTOR inhibition than male podocytes. Furthermore, important novel sex-biased transcription factors were identified in response to mTOR inhibition. This might contribute significantly to sex-different susceptibilities and clinical courses of a variety of sexual dimorphic glomerular kidney diseases.

More specific studies are urgently required, which is additionally supported by the fact that mTOR signaling varies not only with age and tissue, but also by sex, between mouse strains, and between different species [176]. A recent systematic review, targeting aging with rapamycin and its derivatives in humans summarized that positive effects of rapamycin had been shown on the immunological, cardiovascular, and integumentary systems, yet respiratory, digestive, renal and reproductive systems were still underassessed [25].

We do speculate that novel, more specific mTORC1 inhibitors which are already in the pharmaceutical pipeline might elicit beneficial effects in the treatment of chronic kidney diseases especially in male. Furthermore, sex-specific targeting of factors influencing immune functions of the podocyte may contribute to treat immune podocytopathies in the future.

Another interesting aspect is the well-known role of mTOR in aging. Concerning the sex-specific responses towards mTOR inhibition in our study, structural and functional differences in the kidneys under stress might be expected between male and female patients and warrant further clinical investigation.

Future studies will be needed to specifically address pathological disease states or different induction mechanisms and investigate the effect of mTOR inhibition and targeting transcriptional factors to determine the pathophysiological impact of key molecular sex differences elucidated in this study and to move a critical step forward in the era of personalized medicine.

## Conclusions

Our results highlight remarkable intrinsic sex-differences and sex-specific response patterns towards pharmacological challenged podocytes. A large number of known kidney-disease related genes are newly identified to be sexually dimorphic and beyond classical sex hormonal effects further transcription factors were identified in sexually dimorphic response to mTOR inhibition with rapamycin. The female advantage in many kidney diseases` prevalence and disease progression might be strongly influenced by these genomic sex differences. This work can be used as a resource for specific genes to consider when trying to better understand sex differences in kidney health and disease.

## Supplementary Information

Additional file 1: Methods. Detailed descriptions of all methods used.

Additional file 2: Figure S1. Representative FACS cell sorting. Podocyte cell sorting showed a good separation on Fl1 with separation ratio of about 1:3 from the other glomerular cells.

Additional file 3: Figure S2. Comparing sequencing data to previously published data and exclusion cellular contamination and chromosomal effect on sex-specific gene expression. **a** Univariate linear regression analysis indicated a highly significant positive correlation of log2FC of male versus female commonly described genes of podocytes of this study and young cells of renin lineage (Wang Y et al., *Aging (Albany NY).* 2018; https://doi.org/10.18632/aging.101416). Residual standard error: 2.175 on 19 degrees of freedom (DF), Multiple R-squared: 0.7219, Adjusted R-squared: 0.7072, F-statistic: 49.32 on 1 and 19 DF, *P* value: 1.097e-06. **b** Heatmaps of expression after variance stabilization for cell types marker genes from lists obtained from single cell data (Park J et al., Science 2018 (https://doi.org/10.1126/science.aar2131) indicating overrepresentation and enrichment for sex DE genes in podocytes, but not other major kidney cortex cells. Material from 20 mice were used to generate the heapmaps. **c** Venn diagram showing the number of common or unique genes detected in this and published podocyte datasets (Yellow: data from this study, brown: Borries M et al., *Kidney Int.* 2013 (https://doi.org/10.1038/ki.2012.487), green: Lu Y et al., *Kidney Int.* 2017 (https://doi.org/10.1016/j.kint.2017.04.022), Blue: Kann M et al., *J AM Soc Nephrol.* 2015 (https://doi.org/10.1681/ASN.2014090940), orange: Wang Y et al., *Kidney Int.* 2020 (https://doi.org/10.1016/j.kint.2020.05.052). **d** Heatmap of the average normalized expression by sample and by chromosome showing that sexual dimorphic gene expressions do not relate to sex chromosomes.

Additional file 4: Figure S3. Validation of sexual dimorphic transcriptional differences in podocyte. Transcriptomic data were confirmed with qRT-PCR including podocyte specific-genes from isolated podocytes of additional mice. Criteria for gene selection were intrinsically sexually dimorphic podocyte-specific genes with biological relevance for kidney physiology and disease such as *Slc6a6*, *Arhgap32* and *Tjp*1 as well as genes representing main sexual dimorphic GOs such as for “ribosomal proteins” *Rpl13* and “mitochondria/oxidative phosphorylation” *Cox7a2*. Further genes *Daam2*, *Ctsl* and *Arhgap32* were chosen for their relation to kidney diseases and the fact that they did not only show intrinsic sexual dimorphism but were additionally significantly differently regulated in male after rapamycin treatment. Other criteria for gene selection were transcript level expressions higher than 1000, log2FC differences between at least two experimental groups of 0.5–2.0. **a** qPCR results presented as mRNA expression changes from male and female vehicle podocytes (n = 4–8) in the left panel (*P*-values are indicated below each gene name), right panel shows expression level changes of RNA-seq for respective genes of vehicle groups, all genes were normalized to male vehicle*,* **b** qPCR results presented as mRNA expression changes from male rapamycin and male vehicle podocytes (n = 4–8) in the left panel (*P*-values are indicated below each gene name), right panel shows expression level changes of RNA-seq for respective genes of male rapamycin and vehicle groups, all genes were normalized to male vehicle (c) qPCR results presented as mRNA expression changes from female rapamycin and female vehicle podocytes (n = 5–9) in the left panel (*P*-values are indicated below each gene name), right panel shows expression level changes of RNA-seq for respective genes of female rapamycin and vehicle groups, all genes were normalized to female vehicle*,* Reference genes used for normalization were *Hdgf* and *Ywhaz* genes. Results are expressed as mean ± SEM, Mann-Whitney test was *used for statistical comparisons.*

Additional file 5: Figure S4. Validation of proteomics data. **a** Western blots of selected intrinsically sexually dimorphic podocyte-specific proteins (left panel) and quantification (right panel) (n = 4 in each group) of proteins isolated from male and female podocyte-enriched kidney cortex tissue. All proteins were normalized to GAPDH. Results are expressed as mean ± SEM, Mann-Whitney test was *used for statistical comparisons.* **b** Relative differential expression levels of selected proteins as determined by proteomics and immunoblotting.

Additional file 6: Figure S5. Functional grouping of intrinsic sexually dimorphic podocyte genes related to kidney diseases. Heatmap of the expression levels and log2FC of functional groups of sexually dimorphic genes related to kidney diseases. **a** estrogen target, **b** Transcriptional regulators, **c** Cytoskeleton-related genes, **d** metabolism and protein turnover and **e** signaling pathways/kinases related genes. Blue indicates decreased expression level, white no regulation, red increased gene expression level. (4 biological replicates in each group).

Additional file 7: Figure S6. Rapamycin treatment effect on metabolites in male and female podocyte-enriched kidney cortex tissue. Normalized peak areas of the biological replicates from male and female control and rapamycin-treated mice are individually plotted (male vehicle n = 6; male rapamycin n = 6; female vehicle n = 7; female rapamycin n = 5). The overall mean ± SEM is overlain. Median RSD = 11% for pooled QC, median RSD = 36, 31, 32 and 23 for male control, male rapamycin, female control and female rapamycin, respectively.

Additional file 8: Figure S7. pAMPK and AMPK expression in male and female kidney cortex tissue. Western blots indicating activation levels of AMPK by phosphorylation of AMPK at Thr172 and AMPK expression in podocyte-enriched kidney cortex tissue with densitometric quantifications normalized to AMPK for pAMPK and GAPDH for AMPK for **a** male (n = 7) and female (n = 7) vehicle groups and **b** vehicle (n = 6) and rapamycin (n = 5) in male in the left panel and vehicle (n = 6) and rapamycin (n = 5) in female in the right panel. No statistical differences were detected when all biological replicates shown here were included in the analyses. However, when considering the 4th vehicle replicate in the male vehicle and rapamycin groups as an outlier, mean value of pAMPK/AMPK for vehicle was less compared to rapamycin as visualized in the scatter blot below the western blots.

Additional file 9: Table S1. Kidney functional parameters and phenotypic characterization of male and female vehicle and treatment groups.

Additional file 10: Table S2. Podocyte-specific sequencing data – statistical analyses. Differential expression between male & female vehicle groups. Differential expression between male & female rapamycin-treated groups. Log2FC (before & after shrinkage) and *P*-values for the rapamycin effect differential expression in each sex. The combined *P*-value represents minus the logarithm (base 10) of the product between male & female *P*-values. List of genes that had a statistically significant interaction of rapamycin treatment effect between the sexes.

Additional file 11: Table S3. Podocyte-specific sequencing data – TPMs. Transcripts per million, for each sample. Top 100 intrinsically sexually dimorphic genes (y-chromosomal genes excluded) with DE parameters

Additional file 12: Table S4. Intrinsically sexually dimorphic estrogen target genes.

Additional file 13: Table S5. Intrinsically sexually dimorphic transcriptional regulator genes.

Additional file 14: Table S6. EnrichR analysis (KEGG, Wikipathway, MSigDB Hallmark) of intrinsic sex-DE genes for pathway enrichments in male and female podocytes.

Additional file 15: Table S7. Kidney disease-related genes.

Additional file 16: Table S8. Top hits of GSEA pathway analysis of rapamycin effect in male and female podocytes. Selective GSEA pathway related to inflammation and immune response of rapamycin effect in male and female podocytes.

Additional file 17: Table S9. Ingenuity pathway analysis of rapamycin effect in male and female podocytes.

Additional file 18: Table S10. Immune-related genes with intrinsic sexual dimorphism and regulation by rapamycin. Gene list for sublists preparation derived from Bruno et al., New insights into the immune functions of podocytes: the role of complement. Molecular and Cellular Pediatrics (2023) 10:3, https://doi.org/10.1186/s40348-023-00157-3. Sheet: “intrinsic sexual dimorphism”—DE genes related to immunity in male and female podocytes from vehicle treated mice. Sheet: “rapa effect in each sex” –DE genes related to immunity for rapamycin effect in each sex separately.

Additional file 19: Table S11. Podocyte-specific proteomics data.

Additional file 20: Table S12. Metabolomics data. List of metabolite derivatives and their biological group used for reference search. Example template – project QC sample reporting.

Additional file 21: Table S13. Podocyte isolation information and RNA quality control. Podocyte numbers, RNA quantities and RNA quality control. TPM of immediate early genes.

## Data Availability

Data generated and analyzed during this study are included in this published article and its supplementary information files. Further raw data supporting the findings of this study  are openly available in the NCBI´s Gene Expression Omnibus repository [177], and are accessible through GEO Series accession number GSE276967 [https://www.ncbi.nlm.nih.gov/geo/query/acc.cgi?acc=GSE276967].

## References

[1] Meliambro K, He JC, Campbell KN. Podocyte-targeted therapies—progress and future directions. Nat Rev Nephrol. 2024. 10.1038/s41581-024-00843-z. 38724717 10.1038/s41581-024-00843-z

[2] Boddu R, et al. Unique sex- and age-dependent effects in protective pathways in acute kidney injury. Am J Physiol Renal Physiol. 2017;313(3):F740–55. 28679590 10.1152/ajprenal.00049.2017 PMC5625098

[3] Hosszu A, Fekete A, Szabo AJ. Sex differences in renal ischemia-reperfusion injury. Am J Physiol Renal Physiol. 2020;319(2):F149–54. 32567347 10.1152/ajprenal.00099.2020

[4] Rinn JL, et al. Major molecular differences between mammalian sexes are involved in drug metabolism and renal function. Dev Cell. 2004;6(6):791–800. 15177028 10.1016/j.devcel.2004.05.005

[5] Curtis LM. Sex and Gender Differences in AKI. Kidney360. 2024;5(1):160–7. 37990360 10.34067/KID.0000000000000321 PMC10833607

[6] Carrero JJ, Hecking M, Chesnaye NC, Jager KJ. Sex and gender disparities in the epidemiology and outcomes of chronic kidney disease. Nat Rev Nephrol. 2018;14(3):151–64. 29355169 10.1038/nrneph.2017.181

[7] Chesnaye NC, Carrero JJ, Hecking M, Jager KJ. Differences in the epidemiology, management and outcomes of kidney disease in men and women. Nat Rev Nephrol. 2024;20(1):7–20. 37985869 10.1038/s41581-023-00784-z

[8] Garcia GG, et al. Sex and gender differences in chronic kidney disease and access to care around the globe. Semin Nephrol. 2022;42(2):101–13. 35718358 10.1016/j.semnephrol.2022.04.001

[9] Clotet-Freixas S, et al. Sex differences in kidney metabolism may reflect sex-dependent outcomes in human diabetic kidney disease. Sci Transl Med. 2024;16(737):eabm2090. 38446901 10.1126/scitranslmed.abm2090

[10] Mazure CM, Jones DP. Twenty years and still counting: including women as participants and studying sex and gender in biomedical research. BMC Womens Health. 2015;15:94. 26503700 10.1186/s12905-015-0251-9 PMC4624369

[11] Bond KM, McCarthy MM, Rubin JB, Swanson KR. Molecular omics resources should require sex annotation: a call for action. Nat Methods. 2021;18(6):585–8. 34099934 10.1038/s41592-021-01168-6 PMC8764747

[12] NIH Policy on Sex as a Biological Variable. https://orwh.od.nih.gov/sex-gender/nih-policy-sex-biological-variable. Accessed 20 Nov 2022.

[13] Our priorities-Gender and Diversity. https://www.scienceeurope.org/our-priorities/gender-and-diversity/. Accessed 15 Dec 2022.

[14] Gender equality. https://ec.europa.eu/research/participants/docs/h2020-funding-guide/cross-cutting-issues/gender_en.htm. Accessed 20 Dec 2022.

[15] Sex, gender and diversity in the life sciences. https://www.dfg.de/en/research_funding/principles_dfg_funding/diversity_dimensions/lw/index.html. Accessed 20 Dec 2022.

[16] Bairey Merz CN, et al. Sex and the kidneys: current understanding and research opportunities. Nat Rev Nephrol. 2019;15(12):776–83. 31586165 10.1038/s41581-019-0208-6 PMC7745509

[17] Fantus D, Rogers NM, Grahammer F, Huber TB, Thomson AW. Roles of mTOR complexes in the kidney: implications for renal disease and transplantation. Nat Rev Nephrol. 2016;12(10):587–609. 27477490 10.1038/nrneph.2016.108 PMC6553645

[18] Vollenbroker B, et al. mTOR regulates expression of slit diaphragm proteins and cytoskeleton structure in podocytes. Am J Physiol Renal Physiol. 2009;296(2):F418–26. 19019920 10.1152/ajprenal.90319.2008

[19] Zha D, Wu X. Nutrient sensing, signaling transduction, and autophagy in podocyte injury: implications for kidney disease. J Nephrol. 2023;36(1):17–29. 35704261 10.1007/s40620-022-01365-2

[20] Puelles VG, et al. mTOR-mediated podocyte hypertrophy regulates glomerular integrity in mice and humans. JCI Insight. 2019;4(18):e99271. https://insight.jci.org/articles/view/99271 31534053 10.1172/jci.insight.99271 PMC6795295

[21] Ganesh SK, Subathra DC. Molecular and therapeutic insights of rapamycin: a multi-faceted drug from *Streptomyces**hygroscopicus*. Mol Biol Rep. 2023;50(4):3815–33. 36696023 10.1007/s11033-023-08283-x PMC9875782

[22] Weichhart T. mTOR as regulator of lifespan, aging, and cellular senescence: a mini-review. Gerontology. 2018;64(2):127–34. 29190625 10.1159/000484629 PMC6089343

[23] Papadopoli D, et al. AmTOR as a central regulator of lifespan and aging. F1000Res. 2019;8:998. 10.12688/f1000research.17196.1 PMC6611156 31316753

[24] Mota-Martorell N, Jove M, Pamplona R. mTOR complex 1 content and regulation is adapted to animal longevity. Int J Mol Sci. 2022;23(15):8747. 35955882 10.3390/ijms23158747 PMC9369240

[25] Lee DJW, Hodzic Kuerec A, Maier AB. Targeting ageing with rapamycin and its derivatives in humans: a systematic review. Lancet Healthy Longev. 2024;5(2):e152–62. 38310895 10.1016/S2666-7568(23)00258-1

[26] Sharp ZD, Strong R. Rapamycin, the only drug that has been consistently demonstrated to increase mammalian longevity. An update Exp Gerontol. 2023;176: 112166. 37011714 10.1016/j.exger.2023.112166 PMC10868408

[27] Juricic P, et al. Long-lasting geroprotection from brief rapamycin treatment in early adulthood by persistently increased intestinal autophagy. Nat Aging. 2022;2(9):824–36. 37118497 10.1038/s43587-022-00278-w PMC10154223

[28] Konopka AR, Lamming DW, Investigators RP, Investigators E. Blazing a trail for the clinical use of rapamycin as a geroprotecTOR. Geroscience. 2023;45(5):2769–83. 37801202 10.1007/s11357-023-00935-x PMC10643772

[29] Gurgen D, et al. Sex-specific mTOR signaling determines sexual dimorphism in myocardial adaptation in normotensive DOCA-salt model. Hypertension. 2013;61(3):730–6. 23339165 10.1161/HYPERTENSIONAHA.111.00276

[30] Gui Y, Dai C. mTOR signaling in kidney diseases. Kidney360. 2020;1(11):1319–27. 35372878 10.34067/KID.0003782020 PMC8815517

[31] Boerries M, et al. Molecular fingerprinting of the podocyte reveals novel gene and protein regulatory networks. Kidney Int. 2013;83(6):1052–64. 23364521 10.1038/ki.2012.487

[32] Percie du Sert N, et al. The ARRIVE guidelines 2.0: updated guidelines for reporting animal research. PLoS Biol. 2020;18(7): e3000410. 32663219 10.1371/journal.pbio.3000410 PMC7360023

[33] Hohne M, et al. Single-nephron proteomes connect morphology and function in proteinuric kidney disease. Kidney Int. 2018;93(6):1308–19. 29530281 10.1016/j.kint.2017.12.012

[34] Dittmayer C, Volcker E, Wacker I, Schroder RR, Bachmann S. Modern field emission scanning electron microscopy provides new perspectives for imaging kidney ultrastructure. Kidney Int. 2018;94(3):625–31. 30143069 10.1016/j.kint.2018.05.017

[35] Fritsche-Guenther R, Bauer A, Gloaguen Y, Lorenz M, Kirwan JA. Modified protocol of harvesting, extraction, and normalization approaches for gas chromatography mass spectrometry-based metabolomics analysis of adherent cells grown under high fetal calf serum conditions. Metabolites. 2019;10(1):2. 31861324 10.3390/metabo10010002 PMC7023238

[36] Rapamune (sirolimus) tablets, for oral use Reference ID: 4087386. In: Administration USFaD, editor. Revised July 2011 ed. 1999.

[37] Park J, et al. Single-cell transcriptomics of the mouse kidney reveals potential cellular targets of kidney disease. Science. 2018;360(6390):758–63. 29622724 10.1126/science.aar2131 PMC6188645

[38] Wang Y, et al. Global transcriptomic changes occur in aged mouse podocytes. Kidney Int. 2020;98(5):1160–73. 32592814 10.1016/j.kint.2020.05.052 PMC7606654

[39] Kann M, et al. Genome-wide analysis of Wilms’ tumor 1-controlled gene expression in podocytes reveals key regulatory mechanisms. J Am Soc Nephrol. 2015;26(9):2097–104. 25636411 10.1681/ASN.2014090940 PMC4552122

[40] Lu Y, et al. Genome-wide identification of genes essential for podocyte cytoskeletons based on single-cell RNA sequencing. Kidney Int. 2017;92(5):1119–29. 28709640 10.1016/j.kint.2017.04.022

[41] Grath S, Parsch J. Sex-biased gene expression. Annu Rev Genet. 2016;50:29–44. 27574843 10.1146/annurev-genet-120215-035429

[42] Naqvi S, et al. Conservation, acquisition, and functional impact of sex-biased gene expression in mammals. Science. 2019;365(6450):eaaw7317. 31320509 10.1126/science.aaw7317 PMC6896219

[43] Rinn JL, Snyder M. Sexual dimorphism in mammalian gene expression. Trends Genet. 2005;21(5):298–305. 15851067 10.1016/j.tig.2005.03.005

[44] Jassal B, et al. The reactome pathway knowledgebase. Nucleic Acids Res. 2020;48(D1):D498–503. 31691815 10.1093/nar/gkz1031 PMC7145712

[45] Nishi K, Fu W, Kiyama R. Novel estrogen-responsive genes (ERGs) for the evaluation of estrogenic activity. PLoS ONE. 2022;17(8): e0273164. 35976950 10.1371/journal.pone.0273164 PMC9385026

[46] Williams TM, Carroll SB. Genetic and molecular insights into the development and evolution of sexual dimorphism. Nat Rev Genet. 2009;10(11):797–804. 19834484 10.1038/nrg2687

[47] Canadas-Garre M, et al. Genetic susceptibility to chronic kidney disease—some more pieces for the heritability puzzle. Front Genet. 2019;10:453. 31214239 10.3389/fgene.2019.00453 PMC6554557

[48] Li AS, Ingham JF, Lennon R. Genetic disorders of the glomerular filtration barrier. Clin J Am Soc Nephrol. 2020;15(12):1818–28. 32205319 10.2215/CJN.11440919 PMC7769017

[49] Reiser J, Altintas MM. Podocytes. F1000Res. 2016;5:114. 10.12688/f1000research.7255.1 PMC4755401 26918173

[50] Kopp JB, et al. Podocytopathies. Nat Rev Dis Primers. 2020;6(1):68. 32792490 10.1038/s41572-020-0196-7 PMC8162925

[51] Faul C, Asanuma K, Yanagida-Asanuma E, Kim K, Mundel P. Actin up: regulation of podocyte structure and function by components of the actin cytoskeleton. Trends Cell Biol. 2007;17(9):428–37. 17804239 10.1016/j.tcb.2007.06.006

[52] Ha TS. Genetics of hereditary nephrotic syndrome: a clinical review. Korean J Pediatr. 2017;60(3):55–63. 28392820 10.3345/kjp.2017.60.3.55 PMC5383633

[53] Imasawa T, Rossignol R. Podocyte energy metabolism and glomerular diseases. Int J Biochem Cell Biol. 2013;45(9):2109–18. 23806869 10.1016/j.biocel.2013.06.013

[54] Lowik MM, Groenen PJ, Levtchenko EN, Monnens LA, van den Heuvel LP. Molecular genetic analysis of podocyte genes in focal segmental glomerulosclerosis–a review. Eur J Pediatr. 2009;168(11):1291–304. 19562370 10.1007/s00431-009-1017-x PMC2745545

[55] Muller-Deile J, Schiffer M. The podocyte power-plant disaster and its contribution to glomerulopathy. Front Endocrinol (Lausanne). 2014;5:209. 25566185 10.3389/fendo.2014.00209 PMC4266017

[56] Perico L, Conti S, Benigni A, Remuzzi G. Podocyte-actin dynamics in health and disease. Nat Rev Nephrol. 2016;12(11):692–710. 27573725 10.1038/nrneph.2016.127

[57] Rinschen MM, Huesgen PF, Koch RE. The podocyte protease web: uncovering the gatekeepers of glomerular disease. Am J Physiol Renal Physiol. 2018;315(6):F1812–6. 30230368 10.1152/ajprenal.00380.2018

[58] Stelzer G, et al. The GeneCards Suite: from gene data mining to disease genome sequence analyses. Curr Protoc Bioinformatics. 2016;54:1. 10.1002/cpbi.5 27322403

[59] Kramer A, Green J, Pollard J Jr, Tugendreich S. Causal analysis approaches in Ingenuity Pathway Analysis. Bioinformatics. 2014;30(4):523–30. 24336805 10.1093/bioinformatics/btt703 PMC3928520

[60] Kirtana R, Manna S, Patra SK. Molecular mechanisms of KDM5A in cellular functions: Facets during development and disease. Exp Cell Res. 2020;396(2): 112314. 33010254 10.1016/j.yexcr.2020.112314

[61] Plch J, Hrabeta J, Eckschlager T. KDM5 demethylases and their role in cancer cell chemoresistance. Int J Cancer. 2019;144(2):221–31. 30246379 10.1002/ijc.31881

[62] Zhou D, et al. RBP2 induces stem-like cancer cells by promoting EMT and is a prognostic marker for renal cell carcinoma. Exp Mol Med. 2016;48(6): e238. 27282106 10.1038/emm.2016.37 PMC4929691

[63] Chen JH, et al. The down-regulation of XBP1, an unfolded protein response effector, promotes acute kidney injury to chronic kidney disease transition. J Biomed Sci. 2022;29(1):46. 35765067 10.1186/s12929-022-00828-9 PMC9241279

[64] Zhang J, et al. Downregulation of XBP1 protects kidney against ischemia-reperfusion injury via suppressing HRD1-mediated NRF2 ubiquitylation. Cell Death Discov. 2021;7(1):44. 33654072 10.1038/s41420-021-00425-z PMC7925512

[65] Fontecha-Barriuso M, et al. The role of PGC-1alpha and mitochondrial biogenesis in kidney diseases. Biomolecules. 2020;10(2):347. 32102312 10.3390/biom10020347 PMC7072614

[66] Zhang C, et al. The Hippo pathway and its correlation with acute kidney injury. Zool Res. 2022;43(5):897–910. 36052554 10.24272/j.issn.2095-8137.2022.110 PMC9486510

[67] Wong JS, Meliambro K, Ray J, Campbell KN. Hippo signaling in the kidney: the good and the bad. Am J Physiol Renal Physiol. 2016;311(2):F241–8. 27194720 10.1152/ajprenal.00500.2015 PMC5005280

[68] Harlander S, et al. Combined mutation in Vhl, Trp53 and Rb1 causes clear cell renal cell carcinoma in mice. Nat Med. 2017;23(7):869–77. 28553932 10.1038/nm.4343 PMC5509015

[69] Chen J, et al. VNN1 contributes to the acute kidney injury-chronic kidney disease transition by promoting cellular senescence via affecting RB1 expression. FASEB J. 2022;36(9): e22472. 35959877 10.1096/fj.202200496RR

[70] Inoue T, et al. Exit from germinal center to become quiescent memory B cells depends on metabolic reprograming and provision of a survival signal. J Exp Med. 2021;218(1): e20200866. 33045065 10.1084/jem.20200866 PMC7555411

[71] Liu X, Liu B, Qi H. Germinal center reaction and output: recent advances. Curr Opin Immunol. 2023;82: 102308. 37018876 10.1016/j.coi.2023.102308

[72] Li YM, et al. Impact of immunosuppressive drugs on circulating Tfh cells in kidney transplant recipients: a pilot study. Transpl Immunol. 2018;46:1–7. 28974433 10.1016/j.trim.2017.09.005

[73] Bruno V, Muhlig AK, Oh J, Licht C. New insights into the immune functions of podocytes: the role of complement. Mol Cell Pediatr. 2023;10(1):3. 37059832 10.1186/s40348-023-00157-3 PMC10104987

[74] Haydak J, Azeloglu EU. Role of biophysics and mechanobiology in podocyte physiology. Nat Rev Nephrol. 2024;20(6):371–85. 38443711 10.1038/s41581-024-00815-3 PMC12103212

[75] Reiser J, Sever S. Podocyte biology and pathogenesis of kidney disease. Annu Rev Med. 2013;64:357–66. 23190150 10.1146/annurev-med-050311-163340 PMC3736800

[76] Guneykaya D, et al. Transcriptional and translational differences of microglia from male and female brains. Cell Rep. 2018;24(10):2773-83 e6. 30184509 10.1016/j.celrep.2018.08.001

[77] Battaglioni S, Benjamin D, Walchli M, Maier T, Hall MN. mTOR substrate phosphorylation in growth control. Cell. 2022;185(11):1814–36. 35580586 10.1016/j.cell.2022.04.013

[78] Lorenzo HK, Ollero M. Editorial: molecular and cellular biology of podocytes. Front Cell Dev Biol. 2022;10:1037931. 36299489 10.3389/fcell.2022.1037931 PMC9590409

[79] Peres RAS, et al. Rapamycin treatment induces tubular proteinuria: role of megalin-mediated protein reabsorption. Front Pharmacol. 2023;14:1194816. 37484026 10.3389/fphar.2023.1194816 PMC10359992

[80] Kim BS, et al. Reduction of slit diaphragm-associated molecules by sirolimus. Is it enough to induce proteinuria? Transpl Proc. 2017;49(5):1165–9. 10.1016/j.transproceed.2017.03.021 28583549

[81] Zhang J, et al. Rapamycin treatment alleviates chronic GVHD-induced lupus nephritis in mice by recovering IL-2 production and regulatory T cells while inhibiting effector T cells activation. Biomedicines. 2023;11(3):949. 36979928 10.3390/biomedicines11030949 PMC10045991

[82] Tao Y, et al. Sex and strain differences in renal hemodynamics in mice. Physiol Rep. 2023;11(6): e15644. 36946063 10.14814/phy2.15644 PMC10031302

[83] Li A, et al. Rapamycin treatment dose-dependently improves the cystic kidney in a new ADPKD mouse model via the mTORC1 and cell-cycle-associated CDK1/cyclin axis. J Cell Mol Med. 2017;21(8):1619–35. 28244683 10.1111/jcmm.13091 PMC5543471

[84] Kalucki SA, et al. Reference values and sex differences in absolute and relative kidney size. A Swiss autopsy study. BMC Nephrol. 2020;21(1):289. 32689967 10.1186/s12882-020-01946-y PMC7372852

[85] Jean-Faucher C, et al. Sex-related differences in renal size in mice: ontogeny and influence of neonatal androgens. J Endocrinol. 1987;115(2):241–6. 2963887 10.1677/joe.0.1150241

[86] Yang M, et al. Inflammation is more sensitive than cell proliferation in response to rapamycin treatment in polycystic kidney disease. Kidney Blood Press Res. 2024;49(1):60–8. 38167222 10.1159/000535750

[87] Sakaguchi M, et al. Inhibition of mTOR signaling with rapamycin attenuates renal hypertrophy in the early diabetic mice. Biochem Biophys Res Commun. 2006;340(1):296–301. 16364254 10.1016/j.bbrc.2005.12.012

[88] Rangan GK, Coombes JD. Renoprotective effects of sirolimus in non-immune initiated focal segmental glomerulosclerosis. Nephrol Dial Transplant. 2007;22(8):2175–82. 17550925 10.1093/ndt/gfm191

[89] Hu F, Xu Y, Liu F. Hypothalamic roles of mTOR complex I: integration of nutrient and hormone signals to regulate energy homeostasis. Am J Physiol Endocrinol Metab. 2016;310(11):E994–1002. 27166282 10.1152/ajpendo.00121.2016 PMC4935144

[90] Muta K, Morgan DA, Rahmouni K. The role of hypothalamic mTORC1 signaling in insulin regulation of food intake, body weight, and sympathetic nerve activity in male mice. Endocrinology. 2015;156(4):1398–407. 25574706 10.1210/en.2014-1660 PMC4399321

[91] Reifsnyder PC, et al. Rapamycin/metformin co-treatment normalizes insulin sensitivity and reduces complications of metabolic syndrome in type 2 diabetic mice. Aging Cell. 2022;21(9): e13666. 35986566 10.1111/acel.13666 PMC9470898

[92] Xu G, et al. Gastric mammalian target of rapamycin signaling regulates ghrelin production and food intake. Endocrinology. 2009;150(8):3637–44. 19406939 10.1210/en.2009-0372 PMC2717890

[93] Sun J, Langer WJ, Devish K, Lane PH. Compensatory kidney growth in estrogen receptor-alpha null mice. Am J Physiol Renal Physiol. 2006;290(2):F319–23. 16159896 10.1152/ajprenal.00271.2005

[94] Lane PH. Estrogen receptors in the kidney: lessons from genetically altered mice. Gend Med. 2008;5(1):S11–8. 18395676 10.1016/j.genm.2008.03.003

[95] Mank JE, Nam K, Brunstrom B, Ellegren H. Ontogenetic complexity of sexual dimorphism and sex-specific selection. Mol Biol Evol. 2010;27(7):1570–8. 20142440 10.1093/molbev/msq042

[96] Clodfelter KH, et al. Role of STAT5a in regulation of sex-specific gene expression in female but not male mouse liver revealed by microarray analysis. Physiol Genomics. 2007;31(1):63–74. 17536022 10.1152/physiolgenomics.00055.2007 PMC2586676

[97] Lopes-Ramos CM, et al. Sex differences in gene expression and regulatory networks across 29 human tissues. Cell Rep. 2020;31(12): 107795. 32579922 10.1016/j.celrep.2020.107795 PMC7898458

[98] Olsan EE, et al. Signal transducer and activator of transcription-6 (STAT6) inhibition suppresses renal cyst growth in polycystic kidney disease. Proc Natl Acad Sci U S A. 2011;108(44):18067–72. 22025716 10.1073/pnas.1111966108 PMC3207695

[99] Li J, et al. STAT6 contributes to renal fibrosis by modulating PPARalpha-mediated tubular fatty acid oxidation. Cell Death Dis. 2022;13(1):66. 35046382 10.1038/s41419-022-04515-3 PMC8770798

[100] Liu Y, Yu Y, Zhang J, Wang C. The therapeutic effect of dexmedetomidine on protection from renal failure via inhibiting KDM5A in lipopolysaccharide-induced sepsis of mice. Life Sci. 2019;239: 116868. 31682847 10.1016/j.lfs.2019.116868

[101] Kumar A, et al. Reduction in H3K4me patterns due to aberrant expression of methyltransferases and demethylases in renal cell carcinoma: prognostic and therapeutic implications. Sci Rep. 2019;9(1):8189. 31160694 10.1038/s41598-019-44733-y PMC6546756

[102] Yang GJ, et al. The emerging role of KDM5A in human cancer. J Hematol Oncol. 2021;14(1):30. 33596982 10.1186/s13045-021-01041-1 PMC7888121

[103] Chernov AV, Shubayev VI. Sexual dimorphism of early transcriptional reprogramming in degenerating peripheral nerves. Front Mol Neurosci. 2022;15:1029278. 36385770 10.3389/fnmol.2022.1029278 PMC9648404

[104] Joye DAM, Evans JA. Sex differences in daily timekeeping and circadian clock circuits. Semin Cell Dev Biol. 2022;126:45–55. 33994299 10.1016/j.semcdb.2021.04.026 PMC8589873

[105] Nicolaides NC, Chrousos GP. Sex differences in circadian endocrine rhythms: clinical implications. Eur J Neurosci. 2020;52(1):2575–85. 32012359 10.1111/ejn.14692

[106] Lim AS, et al. Sex difference in daily rhythms of clock gene expression in the aged human cerebral cortex. J Biol Rhythms. 2013;28(2):117–29. 23606611 10.1177/0748730413478552 PMC3774838

[107] Ansermet C, et al. The intrinsic circadian clock in podocytes controls glomerular filtration rate. Sci Rep. 2019;9(1):16089. 31695128 10.1038/s41598-019-52682-9 PMC6838779

[108] Miyake Y, et al. Upregulation of OASIS/CREB3L1 in podocytes contributes to the disturbance of kidney homeostasis. Commun Biol. 2022;5(1):734. 35869269 10.1038/s42003-022-03709-x PMC9307819

[109] O’Reilly J, et al. Sex differences in skeletal muscle revealed through fiber type, capillarity, and transcriptomics profiling in mice. Physiol Rep. 2021;9(18): e15031. 34545692 10.14814/phy2.15031 PMC8453262

[110] Liu D, et al. Identification of key genes and candidated pathways in human autosomal dominant polycystic kidney disease by bioinformatics analysis. Kidney Blood Press Res. 2019;44(4):533–52. 31330507 10.1159/000500458

[111] Schneider R, et al. DAAM2 variants cause nephrotic syndrome via actin dysregulation. Am J Hum Genet. 2020;107(6):1113–28. 33232676 10.1016/j.ajhg.2020.11.008 PMC7820625

[112] Schwalm S, et al. Sphingosine kinase-2 deficiency ameliorates kidney fibrosis by up-regulating Smad7 in a MOUSE MODEL OF UNILATERAL URETERAL OBSTRUCTION. Am J Pathol. 2017;187(11):2413–29. 28807595 10.1016/j.ajpath.2017.06.017

[113] Sistani L, et al. Pdlim2 is a novel actin-regulating protein of podocyte foot processes. Kidney Int. 2011;80(10):1045–54. 21814175 10.1038/ki.2011.231

[114] Nephroseq Database. http://www.v5.nephroseq.org. Accessed 20 April 2022.

[115] Blaine J, Dylewski J. Regulation of the actin cytoskeleton in podocytes. Cells. 2020;9(7):1700. 32708597 10.3390/cells9071700 PMC7408282

[116] Sachs N, et al. Blood pressure influences end-stage renal disease of Cd151 knockout mice. J Clin Invest. 2012;122(1):348–58. 22201679 10.1172/JCI58878 PMC3248294

[117] Allison SJ. Basic research: CD151-role in strengthening podocyte-GBM binding. Nat Rev Nephrol. 2012;8(3):132. 22269977 10.1038/nrneph.2012.11

[118] Ju W, et al. Defining cell-type specificity at the transcriptional level in human disease. Genome Res. 2013;23(11):1862–73. 23950145 10.1101/gr.155697.113 PMC3814886

[119] Gujarati NA, Vasquez JM, Bogenhagen DF, Mallipattu SK. The complicated role of mitochondria in the podocyte. Am J Physiol Renal Physiol. 2020;319(6):F955–65. 33073585 10.1152/ajprenal.00393.2020 PMC7792691

[120] Garsen M, et al. Cathepsin L is crucial for the development of early experimental diabetic nephropathy. Kidney Int. 2016;90(5):1012–22. 27575559 10.1016/j.kint.2016.06.035

[121] Altintas MM, et al. Reduction of proteinuria through podocyte alkalinization. J Biol Chem. 2014;289(25):17454–67. 24817115 10.1074/jbc.M114.568998 PMC4067184

[122] Purwaha P, Silva LP, Hawke DH, Weinstein JN, Lorenzi PL. An artifact in LC-MS/MS measurement of glutamine and glutamic acid: in-source cyclization to pyroglutamic acid. Anal Chem. 2014;86(12):5633–7. 24892977 10.1021/ac501451v PMC4063328

[123] Bajwa A, et al. Sphingosine kinase 2 deficiency attenuates kidney fibrosis via IFN-gamma. J Am Soc Nephrol. 2017;28(4):1145–61. 27799486 10.1681/ASN.2016030306 PMC5373443

[124] Imeri F, et al. Loss of sphingosine kinase 2 enhances Wilm’s tumor suppressor gene 1 and nephrin expression in podocytes and protects from streptozotocin-induced podocytopathy and albuminuria in mice. Matrix Biol. 2021;98:32–48. 34015468 10.1016/j.matbio.2021.05.003

[125] Harrison DE, et al. Rapamycin fed late in life extends lifespan in genetically heterogeneous mice. Nature. 2009;460(7253):392–5. 19587680 10.1038/nature08221 PMC2786175

[126] Asadi Shahmirzadi A, et al. Alpha-ketoglutarate, an endogenous metabolite, extends lifespan and compresses morbidity in aging mice. Cell Metab. 2020;32(3):447–56. 32877690 10.1016/j.cmet.2020.08.004 PMC8508957

[127] Rhoads TW, Anderson RM. Alpha-ketoglutarate, the metabolite that regulates aging in mice. Cell Metab. 2020;32(3):323–5. 32877686 10.1016/j.cmet.2020.08.009 PMC8191137

[128] Kaeberlein TL, et al. Evaluation of off-label rapamycin use to promote healthspan in 333 adults. Geroscience. 2023;45(5):2757–68. 37191826 10.1007/s11357-023-00818-1 PMC10187519

[129] https://www.rapamycin.news/t/participate-in-this-new-rapamycin-study/7148. Accessed on 18 July 2024.

[130] Bitto A, et al. Transient rapamycin treatment can increase lifespan and healthspan in middle-aged mice. Elife. 2016;5: e16351. 27549339 10.7554/eLife.16351 PMC4996648

[131] Yamahara K, Yasuda-Yamahara M, Kume S. A novel therapeutic target for kidney diseases: Lessons learned from starvation response. Pharmacol Ther. 2024;254: 108590. 38286162 10.1016/j.pharmthera.2024.108590

[132] Sataranatarajan K, et al. Regulation of elongation phase of mRNA translation in diabetic nephropathy: amelioration by rapamycin. Am J Pathol. 2007;171(6):1733–42. 17991718 10.2353/ajpath.2007.070412 PMC2111098

[133] Inoki K, et al. mTORC1 activation in podocytes is a critical step in the development of diabetic nephropathy in mice. J Clin Invest. 2011;121(6):2181–96. 21606597 10.1172/JCI44771 PMC3104745

[134] Dai H, Thomson AW. The “other” mTOR complex: new insights into mTORC2 immunobiology and their implications. Am J Transplant. 2019;19(6):1614–21. 30801921 10.1111/ajt.15320 PMC6538441

[135] Lamming DW, et al. Rapamycin-induced insulin resistance is mediated by mTORC2 loss and uncoupled from longevity. Science. 2012;335(6076):1638–43. 22461615 10.1126/science.1215135 PMC3324089

[136] Fang Y, et al. The ketone body beta-hydroxybutyrate mitigates the senescence response of glomerular podocytes to diabetic insults. Kidney Int. 2021;100(5):1037–53. 34246657 10.1016/j.kint.2021.06.031 PMC8889914

[137] Tomita I, et al. SGLT2 inhibition mediates protection from diabetic kidney disease by promoting ketone body-induced mTORC1 inhibition. Cell Metab. 2020;32(3):404-19e6. 32726607 10.1016/j.cmet.2020.06.020

[138] Green CL, et al. Dietary restriction of isoleucine increases healthspan and lifespan of genetically heterogeneous mice. Cell Metab. 2023;35(11):1976–95. 37939658 10.1016/j.cmet.2023.10.005 PMC10655617

[139] Jung MJ, et al. Chronic repression of mTOR complex 2 induces changes in the gut microbiota of diet-induced obese mice. Sci Rep. 2016;6:30887. 27471110 10.1038/srep30887 PMC4965768

[140] Simcox J, Lamming DW. The central moTOR of metabolism. Dev Cell. 2022;57(6):691–706. 35316619 10.1016/j.devcel.2022.02.024 PMC9004513

[141] Janes MR, Fruman DA. Immune regulation by rapamycin: moving beyond T cells. Sci Signal. 2009;2(67):pe25. 19383976 10.1126/scisignal.267pe25

[142] O’Shea AE, et al. Immunologic and dose dependent effects of rapamycin and its evolving role in chemoprevention. Clin Immunol. 2022;245: 109095. 35973640 10.1016/j.clim.2022.109095

[143] Shepherd R, Cheung AS, Pang K, Saffery R, Novakovic B. Sexual Dimorphism in Innate Immunity: The Role of Sex Hormones and Epigenetics. Front Immunol. 2020;11: 604000. 33584674 10.3389/fimmu.2020.604000 PMC7873844

[144] Marquez EJ, et al. Sexual-dimorphism in human immune system aging. Nat Commun. 2020;11(1):751. 32029736 10.1038/s41467-020-14396-9 PMC7005316

[145] Dunn SE, Perry WA, Klein SL. Mechanisms and consequences of sex differences in immune responses. Nat Rev Nephrol. 2024;20(1):37–55. 37993681 10.1038/s41581-023-00787-w

[146] Forsyth KS, Jiwrajka N, Lovell CD, Toothacre NE, Anguera MC. The conneXion between sex and immune responses. Nat Rev Immunol. 2024;24(7):487–502. 38383754 10.1038/s41577-024-00996-9 PMC11216897

[147] Jiang H, et al. Understanding the podocyte immune responses in proteinuric kidney diseases: from pathogenesis to therapy. Front Immunol. 2023;14:1335936. 38288116 10.3389/fimmu.2023.1335936 PMC10822972

[148] Banas MC, et al. TLR4 links podocytes with the innate immune system to mediate glomerular injury. J Am Soc Nephrol. 2008;19(4):704–13. 18256364 10.1681/ASN.2007040395 PMC2390962

[149] Kamitaki N, et al. Complement genes contribute sex-biased vulnerability in diverse disorders. Nature. 2020;582(7813):577–81. 32499649 10.1038/s41586-020-2277-x PMC7319891

[150] Gaya da Costa M, et al. Age and sex-associated changes of complement activity and complement levels in a healthy Caucasian population. Front Immunol. 2018;9:2664. 30515158 10.3389/fimmu.2018.02664 PMC6255829

[151] Gal-Oz ST, et al. ImmGen report: sexual dimorphism in the immune system transcriptome. Nat Commun. 2019;10(1):4295. 31541153 10.1038/s41467-019-12348-6 PMC6754408

[152] Doublier S, et al. Testosterone and 17beta-estradiol have opposite effects on podocyte apoptosis that precedes glomerulosclerosis in female estrogen receptor knockout mice. Kidney Int. 2011;79(4):404–13. 20962747 10.1038/ki.2010.398 PMC4775100

[153] Lee HJ, et al. Female protection against diabetic kidney disease is regulated by kidney-specific AMPK activity. Diabetes. 2024;73(7):1167–77. 38656940 10.2337/db23-0807 PMC11189830

[154] Schlosser P, et al. Association of integrated proteomic and metabolomic modules with risk of kidney disease progression. J Am Soc Nephrol. 2024;35(7):923–35. 38640019 10.1681/ASN.0000000000000343 PMC11230725

[155] Herzig S, Shaw RJ. AMPK: guardian of metabolism and mitochondrial homeostasis. Nat Rev Mol Cell Biol. 2018;19(2):121–35. 28974774 10.1038/nrm.2017.95 PMC5780224

[156] Dugan LL, et al. AMPK dysregulation promotes diabetes-related reduction of superoxide and mitochondrial function. J Clin Invest. 2013;123(11):4888–99. 24135141 10.1172/JCI66218 PMC3809777

[157] Smiles WJ, et al. New developments in AMPK and mTORC1 cross-talk. Essays Biochem. 2024. 10.1042/EBC20240007. 38994736 10.1042/EBC20240007 PMC12055038

[158] Lee HJ, et al. Proximal tubular epithelial insulin receptor mediates high-fat diet-induced kidney injury. JCI Insight. 2021;6(3): e143619. 33400689 10.1172/jci.insight.143619 PMC7934847

[159] Ozawa S, et al. Glycolysis, but not Mitochondria, responsible for intracellular ATP distribution in cortical area of podocytes. Sci Rep. 2015;5:18575. 26677804 10.1038/srep18575 PMC4683464

[160] Imasawa T, et al. High glucose repatterns human podocyte energy metabolism during differentiation and diabetic nephropathy. FASEB J. 2017;31(1):294–307. 27825100 10.1096/fj.201600293r PMC5161522

[161] Brinkkoetter PT, et al. Anaerobic glycolysis maintains the glomerular filtration barrier independent of mitochondrial metabolism and dynamics. Cell Rep. 2019;27(5):1551–66. 31042480 10.1016/j.celrep.2019.04.012 PMC6506687

[162] Li SY, et al. Increasing the level of peroxisome proliferator-activated receptor gamma coactivator-1alpha in podocytes results in collapsing glomerulopathy. JCI Insight. 2017;2(14): e92930. 28724797 10.1172/jci.insight.92930 PMC5518556

[163] Yuan Q, et al. Role of pyruvate kinase M2-mediated metabolic reprogramming during podocyte differentiation. Cell Death Dis. 2020;11(5):355. 32393782 10.1038/s41419-020-2481-5 PMC7214446

[164] Chen N, et al. Carbohydrate response element-binding protein regulates lipid metabolism via mTOR complex1 in diabetic nephropathy. J Cell Physiol. 2021;236(1):625–40. 32583421 10.1002/jcp.29890

[165] Ventura-Clapier R, et al. Mitochondria: a central target for sex differences in pathologies. Clin Sci (Lond). 2017;131(9):803–22. 28424375 10.1042/CS20160485

[166] Chella Krishnan K, et al. Sex-specific genetic regulation of adipose mitochondria and metabolic syndrome by Ndufv2. Nat Metab. 2021;3(11):1552–68. 34697471 10.1038/s42255-021-00481-w PMC8909918

[167] Rachakatla A, Kalashikam RR. Calorie restriction-regulated molecular pathways and its impact on various age groups: an overview. DNA Cell Biol. 2022;41(5):459–68. 35451872 10.1089/dna.2021.0922

[168] Korybalska K, Kawka E, Breborowicz A, Witowski J. The role of mTOR inhibitors and HMG-CoA reductase inhibitors on young and old endothelial cell functions, critical for re-endothelialisation after percutaneous coronary intervention: an in vitro study. J Physiol Pharmacol. 2017;68(3):397–405. 28820396

[169] Shindyapina AV, et al. Rapamycin treatment during development extends life span and health span of male mice and Daphnia magna. Sci Adv. 2022;8(37):eabo5482. 36112674 10.1126/sciadv.abo5482 PMC9481125

[170] Ma M, et al. Metformin combined with rapamycin ameliorates podocyte injury in idiopathic membranous nephropathy through the AMPK/mTOR signaling pathway. J Cell Commun Signal. 2023;17(4):1405–15. 37702819 10.1007/s12079-023-00781-8 PMC10713903

[171] Lv F, et al. CD36 aggravates podocyte injury by activating NLRP3 inflammasome and inhibiting autophagy in lupus nephritis. Cell Death Dis. 2022;13(8):729. 35999224 10.1038/s41419-022-05179-9 PMC9399182

[172] Tian Y, et al. Nestin protects podocyte from injury in lupus nephritis by mitophagy and oxidative stress. Cell Death Dis. 2020;11(5):319. 32371936 10.1038/s41419-020-2547-4 PMC7200703

[173] Cheng Q, et al. Caspase-11/4 and gasdermin D-mediated pyroptosis contributes to podocyte injury in mouse diabetic nephropathy. Acta Pharmacol Sin. 2021;42(6):954–63. 32968210 10.1038/s41401-020-00525-z PMC8149386

[174] Ransick A, et al. Single-cell profiling reveals sex, lineage, and regional diversity in the mouse kidney. Dev Cell. 2019;51(3):399-413 e7. 31689386 10.1016/j.devcel.2019.10.005 PMC6948019

[175] Wang Y, et al. Sex differences in transcriptomic profiles in aged kidney cells of renin lineage. Aging (Albany NY). 2018;10(4):606–21. 29676999 10.18632/aging.101416 PMC5940125

[176] Baar EL, Carbajal KA, Ong IM, Lamming DW. Sex- and tissue-specific changes in mTOR signaling with age in C57BL/6J mice. Aging Cell. 2016;15(1):155–66. 26695882 10.1111/acel.12425 PMC4717274

[177] Edgar R, et al. Gene Expression Omnibus: NCBI gene expression and hybridization array data repository. Nucleic Acids Res. 2002;30(1):207–10. 10.1093/nar/30.1.207. 10.1093/nar/30.1.207 PMC99122 11752295

