# Supplementary material for: Sex-specific molecular signature of mouse podocytes in homeostasis and in response to pharmacological challenge with rapamycin
Source: Biol Sex Differ. 2024 Sep 15;15:72. doi: 10.1186/s13293-024-00647-7 (PMC11404044; doi:10.1186/s13293-024-00647-7)
Supplement: Supplementary file 1 — Additional file 1: Methods. Detailed descriptions of all methods used. [file 13293_2024_647_MOESM1_ESM.docx]

**SUPPLEMENTARY METHODS**

**Animals and mouse model**

Gt(ROSA)26Sortm4(ACTB-tdTomato,-EGFP)Luo/J mice (The Jackson Laboratory, Bar Harbour, Massachusetts, USA) [1] crossed with Tg(NPHS2-cre)295Lbh mice have been reported previously [2] . Mice were kept on a mixed genetic background (Sv129/C57Bl6/ICR). Breeding and genotyping were performed according to standard procedures. Mice were housed in an SPF facility with free access to chow and water and a 12-hr day/night cycle. All animal experiments were conducted according to standards and procedures approved by the local Animal Care and Use Committee (LaGeSo Berlin G0241/2015).

Male and female mice aged 12-18 weeks were treated with rapamycin (LC Laboratories, Woburn, Massachusetts, USA) (1.5mg/kg/BW) injected intraperitoneally every third day over a period of 21 days. Rapamycin stock solutions (20mg/mL) were prepared in Miglyol ® 812 (Caleo; Caesar & Loretz GmbH, Hilden, Germany) and fresh injection solution was prepared immediately prior injection by dissolving stock in Dimethyl sulfoxide (DMSO) to working concentration (1:100). Control mice were respectively injected with only DMSO solvent. The size of the groups was determined based on methodological limitations and minimum sample amount required for molecular profiling of single cell podocytes. No distinct statistical test was used to calculate a distinct group size and statistical power. Similar numbers of biological replicates comparable to previously published literature was used. No specific methods were used to randomly allocate samples to groups.

For monitoring of kidney function, 24h urine was collected from metabolic cages at different time points during and at the end of the 21 experiment days. Mice were weighed every third day on the injection day to ensure correct rapamycin dose. Blood serum and cortex kidney sections were harvested after three weeks of treatment. Then mice were sacrificed, and kidneys either flash frozen for histology and metabolomics or were perfused with HBSS buffer prior to podocyte isolation for RNA sequencing and proteomics. Kidney cortex tissue and isolated podocytes were stored at -80°C until further processed. Kidney tissue for histology was subsequently fixed in 4% paraformaldehyde for paraffin embedding for immunostaining,

Serum levels of rapamycin were obtained using LC-MS/MS. Analysis was performed at Labor Berlin (Charité Campus Virchow-Klinikum, Berlin, Germany). Trough concentrations were measured and were within the range of clinically approved therapeutic levels (range 19-26.5 ng/ml) with 24.6 ± 3.5 ng/ml in male and 21.6 ± 1.9 ng/ml in female mice.

**Genotyping**

Genomic DNA was extracted from tail biopsies (≤ 3 millimeter (mm)) for Cre- genotypic confirmation. DNA was amplified with Nphs2: Cre and R26-mTmG alleles using the following primers: Cre- specific primers (Forward primer: 5’ CGTACTGACGGTGGGAGAA 3’, Reverse primer: 5’ CCCGGCAAAACAGGTAGTT 3’) for polymerase chain reactions (PCR)-based genotyping using TaKaRa kit according to manufacturer protocol, with annealing at 57o C for 1.20 minutes. Additionally, mice were tested for mT/mG, DNA was amplified with Tomato specific primers (Forward primer: 5’ CTCTGCTGCCTCCTGGCTTCT 3’), (reverse -wild type 5’ CGAGGCGGATCACAAGCAATA 3’, reverse -mutant type 5’ TCAATGGGCGGGGGTCGTT 3’).

**Kidney function analyses**

Prior to animal sacrifice by cervical dislocation, mice were anaesthetized with isoflurane and blood was collected from orbital sinus, coagulated and centrifuged at 5,000 × g for 10 min to collect serum. 24 h urine samples from the metabolic cages were centrifuged at 5,000 × g for 10 min, supernatant was collected and stored at -80°C for further analysis. Serum creatinine, urea, sodium, glucose, osmolality, albumin and total protein in serum and urine were determined at Labor Berlin (Charité Campus Virchow-Klinikum, Berlin, Germany). Results are presented in Supplementary Table S1 and Figure 1b. One female vehicle mouse had to be excluded due to microalbuminuria.

**Glomeruli and podocyte isolation**

Podocytes were isolated according to Boerries et al., 2013 [2] from 40 mice (eleven vehicle and nine rapamycin treated mice for each sex). In brief, both kidneys were dissected together with the abdominal aorta and perfused manually with a magnetic bead (DynaBeads) solution in Hank’s buffered salt solution (HBSS) at 37°C subsequently followed by perfusion with digestion solution containing collagenase 300 U/mL (Worthington Collagenase Type II, Lakewood, NJ), DNAse 100U/mL (Applichem A3778, Darmstadt, Germany) and pronase 5 IU/mL (Sigma P6911, Schnelldorf, Germany). After mincing both kidneys in a cell culture dish the tissue was further digested for 15min at 37°C under moderate agitation (100 rpm), then digested the tissue was filtered through a 100µm cell strainer on ice. Intact glomeruli from resuspended pellets were separated using a magnetic particle concentrator and further digested at 37°C for 40min at 1400/min to obtain single cells from the glomerular fraction. Magnetic particles and undigested structures were eliminated by sieving the suspension through a 40µm filter and collecting single cells in a conical tube.

FACS sorting to separate GFP-expressing (GFP+) podocytes from GFP-negative (GFP-) glomerular cells and flow cytometry analysis was performed with a BioRad S3 cell sorter (Biorad) with a Laser excitation at 488nm. Only viable cells were sorted (laser excitation 380 nm) and green GFP+ podocyte fractions were directly frozen on dry ice and stored at -80°C for further RNA sequencing and proteomics. Podocytes used for this study displayed a purity of 92-98% as determined by percentage of GFP-positive cells after FACS sorting Supplementary Figure S1. The yields of male and female podocytes are presented in Supplementary Table S12, sheet RNA_quality.

**RNA isolation and quality control**

Total podocyte specific RNA was purified from native podocytes of male and female vehicle- and rapamycin-treated mice Podocytes were separately prepared from each mouse and individual biological replicates subjected to cDNA library preparation and sequencing. After podocyte lysis in Trizol-Ethanol and further purification using Zymo-Spin IC Columns (Zymo Research Europe GmbH, Freiburg, Germany) according to manufacturer´s protocol, RNA was determined by Bioanalyzer runs using Agilent 2100 bioanalyzer. Results proved intact high-quality RNA with RNA integrity number (RIN) 9.2 ± 0.47 in all biological samples (Supplementary Table S13, sheet “RNA_quality”). Primarily 22 mice were allocated to RNA sequencing, however, from one rapamycin-treated male, podocytes could not be isolated due to genotyping error, and a second male vehicle mouse was excluded from sequencing because of low RNA quality. Finally, RNA was sequenced from podocytes of 20 mice (male vehicle = 4; female vehicle n=5; male rapamycin n=5; and female rapamycin n=6). For later podocyte-specific analyses however, one female vehicle mouse [sample ID: f_vehicle_318-S1-RNA1-mRNA_seq1] had to be further excluded when laboratory results were obtained showing micro-albuminuria.

For qPCR experiments, total RNA was extracted from isolated podocytes prepared for RNA sequencing and from podocytes isolated from additional 12 mice (male vehicle n=4; male rapamycin n=2; female vehicle n=3; and female rapamycin n=3) using Quiazol (Qiagen, Hilden, Germany) according to the manufacturer´s protocol. RNA pellets were dissolved in 0.1% DEPC water and the concentration was measured by NanoDrop® Spectrophotometer.

**Transcriptomics (Processing and analysis of RNA-Seq data)**

After generating cDNA libraries from Poly-A selected mRNA (NEB Next Ultra II Directional RNA Library Prep Kit, New England Biolabs, [Massachusetts, United States](https://www.google.com/search?sxsrf=AJOqlzUTrKL0ndrcNpqfN37dZmnsw3JWWA:1676022143266&q=Ipswich,+Massachusetts&si=AEcPFx5y3cpWB8t3QIlw940Bbgd-HLN-aNYSTraERzz0WyAsdCzEadn2wnEJ44zC1xuhoEWLO1NEFVIN49xQQSmJc1aRuAMH9f_h40CvmUMxO6zUaEa2NCWypoQ5bXkf6bQNYLUmnC1794yWPtAa_HYrFaB5FZlDeJ7FabK3Jts6ZaX-WntruMV_8kaag7HzZHnfsBzz2LPE1HMwJZN9H_oWt_dEM05Rmw%3D%3D&sa=X&ved=2ahUKEwjxo_CD1Yr9AhXT7LsIHda_DhcQmxMoAXoECHcQAw)) the obtained DNA was validated with the Agilent TapeStation platform (TapeStation D1000 HS – Kit) and deep RNA sequencing (2x75 per lane) was carried out on an Illumina HiSeq4000 system (Illumina, California, USA). Sequencing performance provided 36-46 million paired-end reads per sample detecting 84% of all transcripts uniquely mapping sequences aligned to only one single gene within the genome.

Sequencing reads were mapped to mm10_GRCm38.p4 using STAR 2.5.0b, intersected with Ensembl GRCm38_mm10 (release 89) gene models using GenomicRanges[3] and counted with the Bioconductor package Rsubread[4]. Normalization and differential expression computation was done using DESeq2[5]. Version 1.26.0. Several types of normalized expression values were used: variance-stabilized values obtained by the vst algorithm (blind mode) implemented in DESeq2 were used for the PCA (500 most varying genes only), and base mean normalized expression over all samples in the model, computed with differential expression, were used for MA plots. Differential expression was computed in separate models that only contained samples from the conditions involved in the contrast of interest. For example, the differential expression results between male and female control animals were computed without samples from treated animals. The interaction model contained all samples, and the interaction term refers to the difference between the treatment effect in males & females. Expression changes of genes were called significant when the *P* value adjusted for multiple correction (Benjamini-Hochberg method) was lower than 0.05. When required, shrinkage was applied to log2-fold changes using the apeglm algorithm[6] version 1.8.0. All normalization and differential expression computations were done on gene models labeled by ENSEMBL gene identifiers. Gene names were obtained from the original ENSEMBL v89 feature set, and from the more recent org.Mm.eg.db Bioconductor package version 3.13.0. As the latter doesn't guarantee one-to-one mapping between identifiers, the feature with the highest average expression was selected, when a gene symbol was matched by more than one ENSEMBL. The Bioconductor package clusterProfiler[7] version 3.14.3 was used to assess over-representation and enrichment of Gene Ontology terms available from the Bioconductor package GO.db version 3.10.0. TPMs were computed from the read counts over the genes, using the gene's total exonic length for the normalization by length (Supplementary Table S3).

**Expression ranks for immediate early genes**

To assess possible differences in stress reaction from cell isolation between the different experimental groups, the dataset was filtered for the immediate early genes, *Fos, Jun, Egr1* and *Nr4a1*. None of them were significantly differently expressed between vehicle groups or treatment effects in each sex. Supplementary Table S13, sheet TPM_immediate_early_genes.

**Quantitative Real-Time PCR analysis**

1µg of total RNA was reverse-transcribed to cDNA by M MuLV Reverse Transcriptase (Thermo fisher science, Germany) using Oligo dT primer, according to the manufactures recommendation.

Specific oligonucleotide primers for *Slc6a6, Tjp1*, *Daam2*, *Ctsl*, *Arhgap32*, *Cox7a2* and *Rpl13* were designed using the database of the National Center for Biotechnology Information (NCBI) (see table below) and the quantitative real time PCR was conducted on a ABI® 7500 Fast Real Time PCR system (Applied Biosystems, Foster City, CA, USA) using SYBER GREEN (Thermo fisher science, Germany)Each amplification reaction was performed in duplicates and relative mRNA expression levels were normalized to *Hdgf* and *Ywazh*. The primer sequences were synthesized by Biolegio, Netherlands at a concentration of 100 μM and were diluted to 10 μM in ultra-pure water.

The following primers were used for qPCR:

| Sequences Gene | Forward primer ( 5’ 3’ ) | Reverse primer ( 5’ 3’ ) |
| --- | --- | --- |
| *Slc6a6* | AAAATGGTGGAGGTGCGTTC | ACACAATGACGATGGATGCG |
| *Arhgap32* | TCCGACCTAGAAGACCCAGA | AGTCAACATCCTCAGCCGAA |
| *Tjp1* | CAACAGGTACAGGCCAGAGG | CAGAGGAGGGACAACTGCAG |
| *RPl13* | CCCTCCACCCTATGACAAG | TTCTCCTCCAGAGTGGCTGT |
| *Cox7a2* | GACCATCAGCACCACTTCAC | CTTCTTGGGAAATGCAGCCA |
| *Ctsl* | TGTAGCAGCAAGAACCTCGA | TGTCCCGGTCTTTGGCTATT |
| *Daam2* | AAGCTGCCAAAGTCAACCTG | TCCTGCTCTCCGAAATGTGT |
| *Ywhaz* | TCTGGCCCTCAACTTCTCTG | CCTGCTTCTGCTTCATCTCC |
| *Hdgf* | ACTCCCCTAAACGTCCCAAG | TCTCATGATCTCTGACGCCC |

**Protein sample preparation and proteomics analysis**

Flash-frozen FACS-sorted podocyte pellets (n=3 male vehicle and n=3 female vehicle) were solubilized with 40 *µ*L 8% SDS and heated at 95°C for 5 minutes to solubilize and denature all proteins. Next, 25 *µ*U Benzonase was added to the solution, and samples were reduced with 5 mM Dithiothreitol (DTT) at 37°C for 30 minutes. Then, proteins were alkylated at room temperature with 10 mM Indole-3-Acetic Acid (IAA) in the dark. Proteins were prepared and digested using Trypsin and LysC using the SP3 ultrasensitive proteomics technique as previously described. FACS-sorted podocytes were prepared using SP3-bead preparation as previously described[8] with modifications[9]. Proteomics data acquisition was performed on a quadrupole Orbitrap hybrid mass spectrometer (QExactive Plus, Thermofisher science, Germany) coupled to an easynLC as previously described[10]. Quantitative analysis was performed using MaxQuant. Raw files were searched against a database consisting of a mouse Uniprot reference database without isoforms (downloaded in January 2017) and with common contaminants. Default search parameters were used, including methionine oxidation as a variable modification, cysteine alkylation as a fixed modification. False discovery rate (FDR) was set to 0.01 for peptide spectrum, site, and protein identification using the target decoy approach. Match between run options was enabled. The search option also included removal of all peptides that were identified by one peptide and by post-translational modification only. The label-free quantitative (LFQ) algorithm option was enabled to obtain label-free quantification intensities. Protein expression was further analyzed using Perseus 1.5.5.3 and the 2D-Go enrichment algorithm[11].

Selected sexually dimorphic podocyte-specific protein expression data of proteomics were validated by western blot analyses (Supplementary Figure S4).

**Functional enrichment analysis**

GO annotations were retrieved from EnrichR database[12-14] and PANTHER database[15-17] (accessed February 2021) with use of Mus musculus species genes. KEGG_mus_2019, WikiPathways_mus 2018 and Panther GO-slim (updated in 2020) were used for GO enrichment analyses. Statistical significance was set as P-value <0.05 with an overlap of more than 4 genes compared with background list (10682 genes for vehicle comparison). Moreover, correction for multiple testing by FDR value <0.05 was obtained (for male and female overexpression GOs with cut point <0.1). Gene set enrichment analysis for whole transcriptome for rapamycin effect on both male and female transcriptome was done using GSEA software[18, 19]. Proteomics data biological process GO enrichment analysis were retrieved from EnrichR. All proteins with FC>2 were included in the analysis. Statistical significance was set as P-value<0.05 with an overlap of more than 6 genes compared with background list (2700 proteins). Further analysis for possible sexual dimorphism on rapamycin effect on different activation of canonical pathways and upstream regulators were performed using QIAGEN’s Ingenuity® Pathway Analysis (IPA®, QIAGEN Redwood City, United States)( ww.qiagen.com/ingenuity) tools.

**Western blot analysis**

Kidney cortex tissues was homogenized in RIPA lysis buffer (50 mM TrisHCl pH 8.0, 150 mM sodium chloride, 5 mM EDTA, 1% NP-40, 0,5 % Sodium deoxycholate, 0,1 % SDS with inhibitors (5 mM sodium fluoride, 10 mM β-glycerophosphate, 1 mM sodium orthovanadate_,_ 2.5 mM sodium pyrophosphate)). The homogenization was carried out using mechanical tissue disruptor (MP Biomedical FastPrep 24 Tissue Homogenizer). Protein concentration was determined by protein assay (BioRad laboratories, Munich, Germany). 60 μg of protein was loaded onto 8-12% Bis-Tris polyacrylamide gels and separated by gel electrophoresis, followed by transfer onto Polyvinylidene fluoride (PVDF) membrane (Healthcare, Amersham, UK). Blotting and immunostaining were performed according to standard protocol. The following primary antibodies were used; GAPDH (Hytest 5G4), Phosphorylated P70S6K Thr389 (R&D AF8963), P70S6K (R&D AF8962) pAMPK Thr172 (Cell signaling 2535), AMPK (Cell signaling 2532), Laminin β2 (Santa Cruz (C4) 59980), Nephrin (Abcam 58968), Synaptopodin (Santa Cruz (D-9) 515842), ZO-1 (Invitrogen 61-7300), ACTN4 (Abcam 59468).

All primary antibodies were diluted 1:1000 and incubated overnight at 4°C. 1:15000 and 1:20000 Peroxidase-conjugated AffiniPure Donkey Anti-Rabbit IgG and Anti-mouse secondary antibodies were incubated for 1-2 hours at room temperature (Dianova GmbH, Hamburg, Germany). Bands were visualized with a SuperSignal West Pico Plus (Thermo fisher science, Germany) enhanced chemiluminescence system according to the manufacturer's instructions The development procedure was supported with the digital image system of Syngene G:BOX Chemi XRQ (VWR, Germany). When further antigen detection was required, membranes were incubated in glycine stripping buffer for 30 minutes at 52°C. After further washing cycles and blocking, the membranes were reused for subsequent antibody probes.

All protein lysates for western blots shown in this manuscript (Figure 1f, Figure S5 and Figure S7 were loaded on the same gel for all antibodies of interest, except for pAMPK rapamycin effect in Figure S7, male and female lysate samples were run for each sex on separate gels.

**Histological analyses**

Tissues were processed with standard protocol using Thermo scientific HistoStar^Tm^ A810101000 issue 2 (Thermo fisher science, Germany), and stored at 4°C. Paraffin-blocks were cut into 4 μm-thick sections with a microtome (Thermo fisher science, Germany), transferred to microscope slides and dried over night at room temperature. Paraffin-embedded kidney tissue sections were stained with PAS staining and Picro red Sirius staining according to standard protocol. Images were evaluated in at least 50 glomeruli of each mouse Field of view (FoV) at 200× magnification under a microscope (Zeiss, Jena, Germany).

Further, 4 µm thick Cryo sections were performed from kidney cortex tissue from Cre-positive and Cre-negative NPHS2 mice embedded in O.C.T. and used for fluorescence microscopy to detect genetic labelling and fluorescent co-staining with synaptopodin antibodies. Sections were fixed using 4 % formaldehyde followed by permeabilization with 0.3 % Triton X100. After blocking with normal goat serum, sections were incubated with or without synaptopodin antibody (Santa Cruz (D-9): sc 515842 1:50) overnight at 4°C. Then, sections were incubated for 1-2 hours at room temperature with Alexa FluorTM 633 (Thermo fisher science, Germany). After washing three times with PBS for 5 min, nuclear counterstaining was performed with DAPI for 5 min and then slides were mounted with Aqua polymount mounting medium. Images were evaluated at 200x magnification under a fluorescence microscope (Zeiss, Jena, Germany).

**Electron microscopy**

A total of eight mice (two vehicle and two rapamycin treated mice for each sex) wase used for electron microscopic examination. After three weeks of treatment, mice were anesthetized with ketamine and perfusion-fixed with 3% paraformaldehyde in 0.1M Na-cacodylate buffer applied via catheter inserted in the abdominal aorta. Kidneys were post-fixed with 1.5% glutaraldehyde in PBS/picric acid, embedded in EPON812 (Sigma-Aldrich, Germany), ultrathin-sectioned and examined with a Zeiss EM912 transmission electron microscope as previously described [20].

**Metabolomics**

Metabolomics was performed as described previously [21]. In summary, 50 mg of podocytes-enriched cortex kidney tissue was used from male vehicle (n=6), male rapamycin (n=6), female vehicle (n=7), and female rapamycin (n=5). The tissue was lyzed by addition of 1 mL/50 mg tissue Methanol:Chloroform:Water (5:2:1, v/v/v) mixture containing 2 µg/mL cinnamic acid as internal standard. Samples were extracted using Precellys 24 Tissue Lyser (Peqlab) combined with a nitrogen cooling unit (Cryolys). Samples were incubated for 30 min at 4°C with shaking. 500 µL/50 mg tissue of water was added followed by incubation for 15 min at 4°C with shaking. The vials were centrifuged at 18,213 x g at 4°C. After extraction, 200 µL of polar phases were dried at 30°C at a speed of 1,550 x g at 0.1 mbar using a rotational vacuum concentrator (RVC 2-33 CDplus, Christ, Osterode am Harz, Germany). Samples were pooled after extraction and used as a quality control sample to test the technical variability. They were prepared alongside the samples in the same way.

**Gas chromatography-mass spectrometry (GC-MS) measurement**

All polar extracts were stored dry at -80°C until analysis. Extracts were removed from the freezer and dried in a rotational vacuum concentrator for 60 min before further processing to ensure there was no residual water which may influence derivatization efficiency. Dried extracts were dissolved in 15 µL of methoxyamine hydrochloride solution (40 mg/mL in pyridine) and incubated for 90 min at 30°C with constant shaking, followed by the addition of 50 µL of N-methyl-N-[trimethylsilyl]triﬂuoroacetamide (MSTFA) and incubation at 37°C for 60 min. The extracts were centrifuged for 10 min at 18,213 x g, and aliquots of 25 µL were transferred into glass vials for GC-MS measurement. An identification mixture for reliable compound identification was prepared and derivatized in the same way[22] and an alkane mixture for reliable retention index calculation was included. Metabolite analysis was performed on a Pegasus 4D GCxGC TOFMS-System (LECO Corporation, St. Joseph, MN, USA) complemented with an auto-sampler (Gerstel MPS DualHead with CAS4 injector, Mühlheim an der Ruhr, Germany). The samples were injected in split mode (split 1:5, injection volume 1 µL) in a temperature-controlled injector with a baﬄed glass liner (Gerstel, Mühlheim an der Ruhr, Germany). The following temperature program was applied during sample injection: the column was allowed to equilibrate at 68°C for 2 minutes followed by a first temperature gradient with a rate of increase of 5°C/min until a maximum of 120°C was reached. Subsequently the rate increased to 7 °C/min up to a maximum temperature of 200°C. This was immediately followed by a third gradient of 12°C/min up to a maximum temperature of 320°C with a hold time of 7.5 min. Gas chromatographic separation was performed on an Agilent 7890 (Agilent Technologies, Santa Clara, CA, USA), equipped with a VF-5 ms column (Agilent Technologies, Santa Clara, CA, USA) of 30 m length, 250 µm inner diameter, and 0.25 µm ﬁlm thickness. Helium was used as the carrier gas with a ﬂow rate of 1.2 mL/min. The spectra were recorded in a mass range of 60 to 600 m/z with 10 spectra/sec. The GC-MS chromatograms were processed with the ChromaTOF software (LECO Corporation, St. Joseph, MN, USA) including baseline assessment, peak picking and computation of the area and height of peaks without a calibration by using an in-house created reference and library containing the top 3 masses by intensity for 42 metabolites (55 derivatives) related to the central carbon metabolism (Supplementary Table S12, Sheet “metabolite list”). The data were exported and merged by an *in-house* written R script. The metabolites were considered valid when they appeared in minimum of n=3 biological replicates. Quality control samples were run at the beginning and end of the analyses and every 12 samples. Data were normalized to cinnamic acid (internal standard). Relative quantities were used. Outlier detection was done using GraphPad (https://www.graphpad.com/quickcalcs/Grubbs1.cfm). Wilcoxon rank sum test (*P*-value < 0.05) was used for statistics between the different conditions. The ratios between females and males or rapamycin and control were calculated. For better visualization the log2 from the ratio was generated.

**Statistics**

For quantitative data, statistical tests (Wilcoxon ranked sum test, Kruskal-Wallis test and linear regression using R package tidyverse, R version 4.0.0) and Graph Pad 9 (Prism, San Diego, CA, USA) was used for the statistical analysis. *P*-value<0.05 was considered significant. For large-scale data, correction for multiple testing was performed as described in the respective omics method sections.

ANOVA test was used to compare the four experimental groups and Bonferroni's post hoc tests were carried out when a significant difference was found between the four experimental group. The number of biological replicates and statistical tests used for analysis are further indicated in the figure legends.

**Supplementary References**

1. Muzumdar MD, Tasic B, Miyamichi K, Li L, Luo L. A global double-fluorescent Cre reporter mouse: Genesis. 2007;45(9):593-605.

2. Boerries M, et al. Molecular fingerprinting of the podocyte reveals novel gene and protein regulatory networks: Kidney Int. 2013;83(6):1052-64.

3. Lawrence M, et al. Software for computing and annotating genomic ranges: PLoS Comput Biol. 2013;9(8):e1003118.

4. Liao Y, Smyth GK, Shi W. The Subread aligner: fast, accurate and scalable read mapping by seed-and-vote: Nucleic Acids Res. 2013;41(10):e108.

5. Love MI, Huber W, Anders S. Moderated estimation of fold change and dispersion for RNA-seq data with DESeq2: Genome Biol. 2014;15(12):550.

6. Zhu A, Ibrahim JG, Love MI. Heavy-tailed prior distributions for sequence count data: removing the noise and preserving large differences: Bioinformatics. 2019;35(12):2084-92.

7. Yu G, Wang LG, Han Y, He QY. clusterProfiler: an R package for comparing biological themes among gene clusters: OMICS. 2012;16(5):284-7.

8. Hughes CS, et al. Single-pot, solid-phase-enhanced sample preparation for proteomics experiments: Nat Protoc. 2019;14(1):68-85.

9. Koehler S, et al. Proteome Analysis of Isolated Podocytes Reveals Stress Responses in Glomerular Sclerosis: J Am Soc Nephrol. 2020;31(3):544-59.

10. Hohne M, et al. Single-nephron proteomes connect morphology and function in proteinuric kidney disease: Kidney Int. 2018;93(6):1308-19.

11. Cox J, Mann M. 1D and 2D annotation enrichment: a statistical method integrating quantitative proteomics with complementary high-throughput data: BMC Bioinformatics. 2012;13 Suppl 16:S12.

12. Chen EY, et al. Enrichr: interactive and collaborative HTML5 gene list enrichment analysis tool: BMC Bioinformatics. 2013;14:128.

13. Kuleshov MV, et al. Enrichr: a comprehensive gene set enrichment analysis web server 2016 update: Nucleic Acids Res. 2016;44(W1):W90-7.

14. Xie Z, et al. Gene Set Knowledge Discovery with Enrichr: Curr Protoc. 2021;1(3):e90.

15. Mi H, et al. PANTHER version 16: a revised family classification, tree-based classification tool, enhancer regions and extensive API: Nucleic Acids Res. 2021;49(D1):D394-D403.

16. Mi H, et al. Protocol Update for large-scale genome and gene function analysis with the PANTHER classification system (v.14.0): Nat Protoc. 2019;14(3):703-21.

17. Thomas PD, et al. PANTHER: a library of protein families and subfamilies indexed by function: Genome Res. 2003;13(9):2129-41.

18. Mootha VK, et al. PGC-1alpha-responsive genes involved in oxidative phosphorylation are coordinately downregulated in human diabetes: Nat Genet. 2003;34(3):267-73.

19. Subramanian A, et al. Gene set enrichment analysis: a knowledge-based approach for interpreting genome-wide expression profiles: Proc Natl Acad Sci U S A. 2005;102(43):15545-50.

20. Dittmayer C, Volcker E, Wacker I, Schroder RR, Bachmann S. Modern field emission scanning electron microscopy provides new perspectives for imaging kidney ultrastructure: Kidney Int. 2018;94(3):625-31.

21. Fritsche-Guenther R, Bauer A, Gloaguen Y, Lorenz M, Kirwan JA. Modified Protocol of Harvesting, Extraction, and Normalization Approaches for Gas Chromatography Mass Spectrometry-Based Metabolomics Analysis of Adherent Cells Grown Under High Fetal Calf Serum Conditions: Metabolites. 2019;10(1):2

22. Opialla T, Kempa S, Pietzke M. Towards a More Reliable Identification of Isomeric Metabolites Using Pattern Guided Retention Validation: Metabolites. 2020;10(11):457
