# Supplementary material for: Sex-specific molecular signature of mouse podocytes in homeostasis and in response to pharmacological challenge with rapamycin
Source: Biol Sex Differ. 2024 Sep 15;15:72. doi: 10.1186/s13293-024-00647-7 (PMC11404044; doi:10.1186/s13293-024-00647-7)
Supplement: Supplementary file 2 — Additional file 2: Figure S1. Representative FACS cell sorting. Podocyte cell sorting showed a good separation on Fl1 with separation ratio of about 1:3 from the other glomerular cells. [file 13293_2024_647_MOESM2_ESM.pdf]

Supplementary Figure S1

Representative results from fluorescence-activated cell sorting of glomerular cells

Single cell suspension from isolated glomeruli	Tomato-positive non-podocyte glomerular cells	GFP-positive podocytes

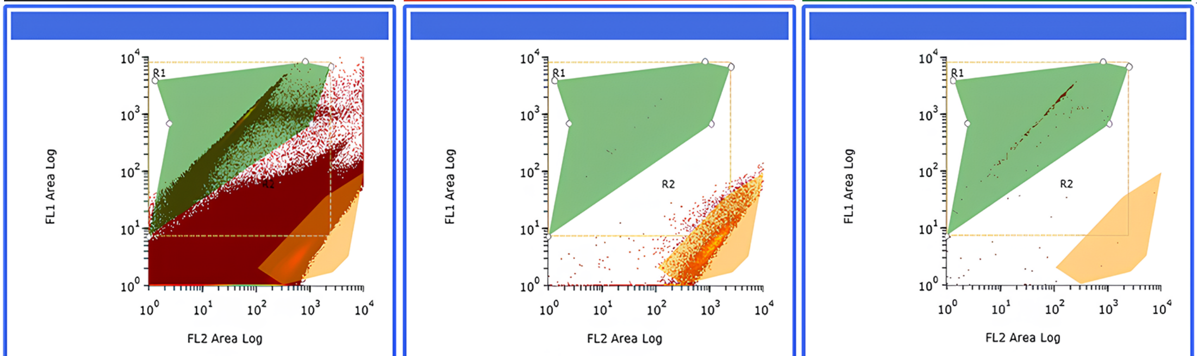

Single cell suspension from isolated glomeruli — Statistics

Label	Count	% of Total	% of Plot
Total	1167252	100.00 %	100.00 %
R2	355227	30.43 %	30.43 %
R1	149799	12.83 %	12.83 %

Tomato-positive non-podocyte glomerular cells — Statistics

Label	Count	% of Total	% of Plot
Total	9164	100.00 %	100.00 %
R2	7940	86.64 %	86.64 %
R1	13	0.14 %	0.14 %

GFP-positive podocytes — Statistics

Label	Count	% of Total	% of Plot
Total	382	100.00 %	100.00 %
R2	5	1.31 %	1.31 %
R1	315	82.46 %	82.46 %

Yield: Average of 300,000 podocytes per mouse with a purity of more than 80 %
