# Supplementary figures and images for: Sex-specific molecular signature of mouse podocytes in homeostasis and in response to pharmacological challenge with rapamycin
Source: Biol Sex Differ. 2024 Sep 15;15:72. doi: 10.1186/s13293-024-00647-7 (PMC11404044; doi:10.1186/s13293-024-00647-7)

Supplementary Figure S2

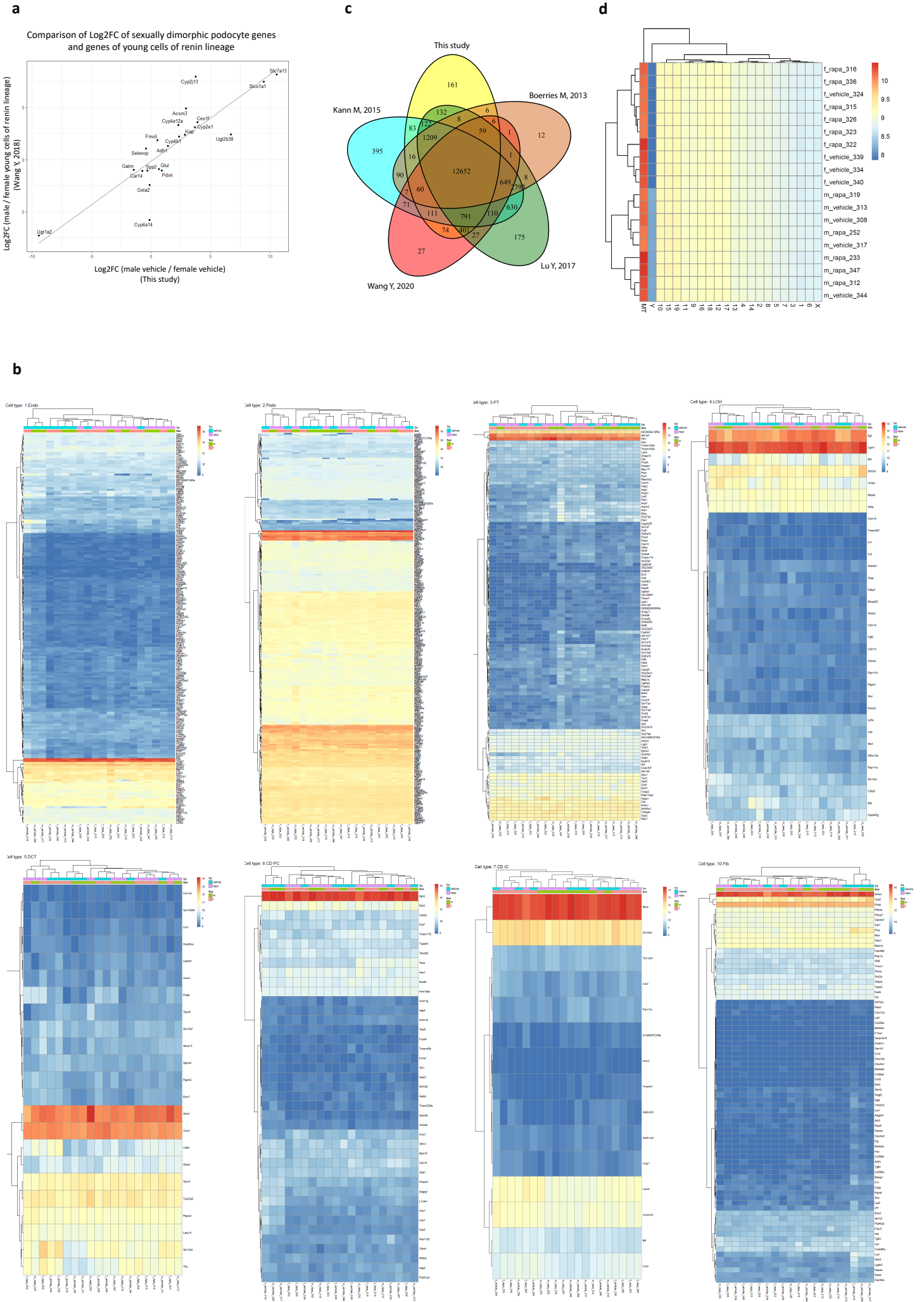

Supplement: Supplementary file 3 — Additional file 3: Figure S2. Comparing sequencing data to previously published data and exclusion cellular contamination and chromosomal effect on sex-specific gene expression. a Univariate linear regression analysis indicated a highly significant positive correlation of log2FC of male versus female commonly described genes of podocytes of this study and young cells of renin lineage (Wang Y et al., Aging (Albany NY). 2018; https://doi.org/10.18632/aging.101416). Residual standard error: 2.175 on 19 degrees of freedom (DF), Multiple R-squared: 0.7219, Adjusted R-squared: 0.7072, F-statistic: 49.32 on 1 and 19 DF, P value: 1.097e-06. b Heatmaps of expression after variance stabilization for cell types marker genes from lists obtained from single cell data (Park J et al., Science 2018 (https://doi.org/10.1126/science.aar2131) indicating overrepresentation and enrichment for sex DE genes in podocytes, but not other major kidney cortex cells. Material from 20 mice were used to generate the heapmaps. c Venn diagram showing the number of common or unique genes detected in this and published podocyte datasets (Yellow: data from this study, brown: Borries M et al., Kidney Int. 2013 (https://doi.org/10.1038/ki.2012.487), green: Lu Y et al., Kidney Int. 2017 (https://doi.org/10.1016/j.kint.2017.04.022), Blue: Kann M et al., J AM Soc Nephrol. 2015 (https://doi.org/10.1681/ASN.2014090940), orange: Wang Y et al., Kidney Int. 2020 (https://doi.org/10.1016/j.kint.2020.05.052). d Heatmap of the average normalized expression by sample and by chromosome showing that sexual dimorphic gene expressions do not relate to sex chromosomes. [file 13293_2024_647_MOESM3_ESM.pdf]

Supplementary Figure S6

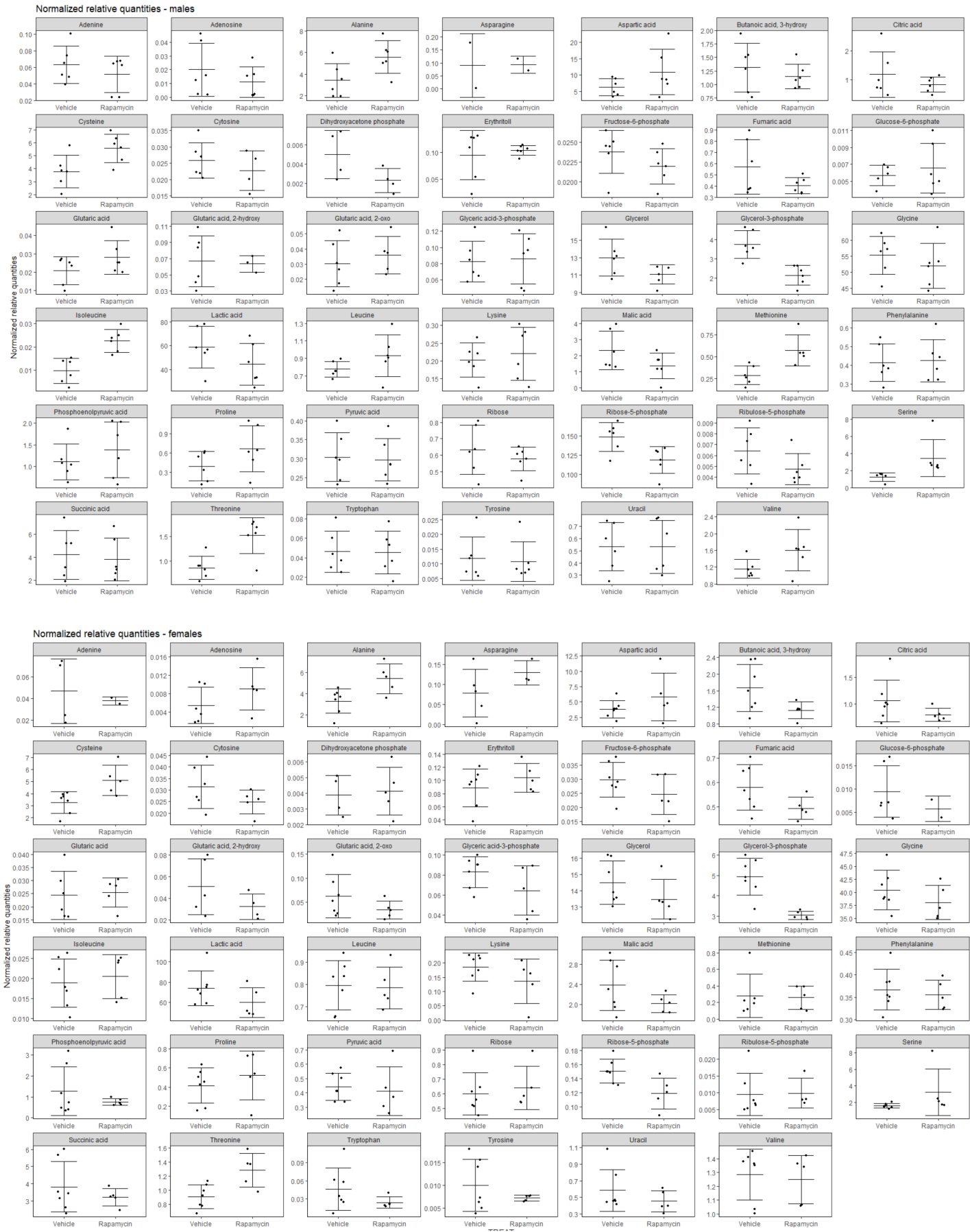

Supplement: Supplementary file 7 — Additional file 7: Figure S6. Rapamycin treatment effect on metabolites in male and female podocyte-enriched kidney cortex tissue. Normalized peak areas of the biological replicates from male and female control and rapamycin-treated mice are individually plotted (male vehicle n = 6; male rapamycin n = 6; female vehicle n = 7; female rapamycin n = 5). The overall mean ± SEM is overlain. Median RSD = 11% for pooled QC, median RSD = 36, 31, 32 and 23 for male control, male rapamycin, female control and female rapamycin, respectively. [file 13293_2024_647_MOESM7_ESM.pdf]
