# Supplementary material for: Sex-specific molecular signature of mouse podocytes in homeostasis and in response to pharmacological challenge with rapamycin
Source: Biol Sex Differ. 2024 Sep 15;15:72. doi: 10.1186/s13293-024-00647-7 (PMC11404044; doi:10.1186/s13293-024-00647-7)

Supplementary Figure S3

RNA-seq data validation for selected intrinsically significantly DE genes

a RNA-seq data validation for selected intrinsically significantly DE genes

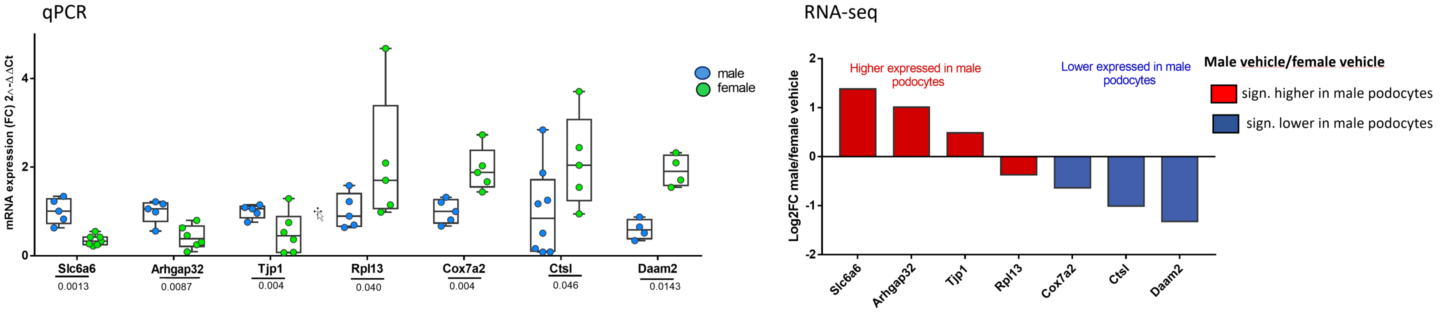

b RNA-seq data validation for selected DE genes in response to rapamycin treatment in male

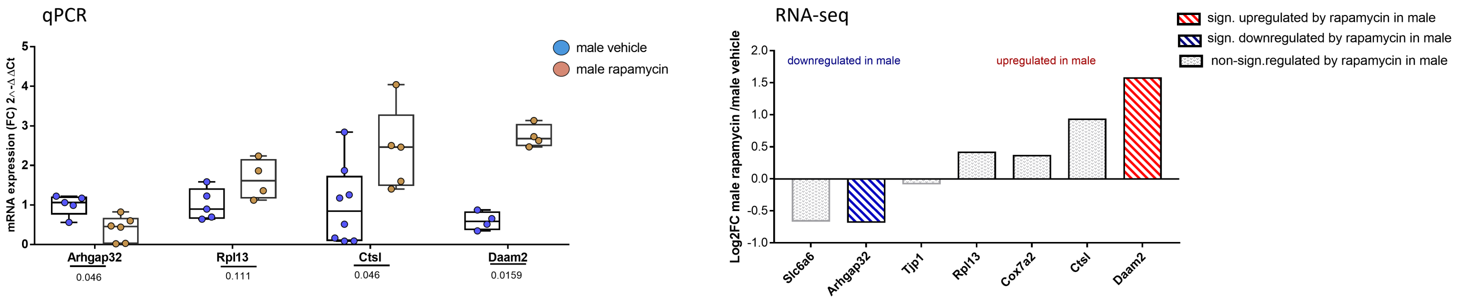

c RNA-seq data validation for selected genes in response to rapamycin treatment in female

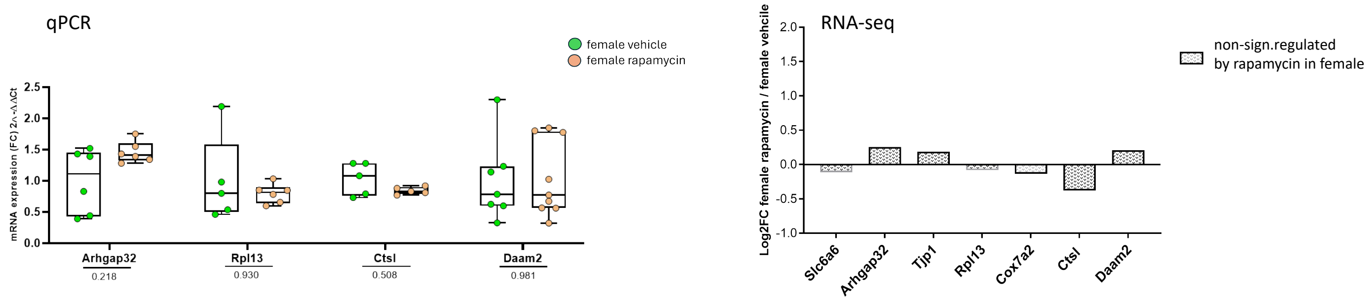

Supplement: Supplementary file 4 — Additional file 4: Figure S3. Validation of sexual dimorphic transcriptional differences in podocyte. Transcriptomic data were confirmed with qRT-PCR including podocyte specific-genes from isolated podocytes of additional mice. Criteria for gene selection were intrinsically sexually dimorphic podocyte-specific genes with biological relevance for kidney physiology and disease such as Slc6a6, Arhgap32 and Tjp1 as well as genes representing main sexual dimorphic GOs such as for “ribosomal proteins” Rpl13 and “mitochondria/oxidative phosphorylation” Cox7a2. Further genes Daam2, Ctsl and Arhgap32 were chosen for their relation to kidney diseases and the fact that they did not only show intrinsic sexual dimorphism but were additionally significantly differently regulated in male after rapamycin treatment. Other criteria for gene selection were transcript level expressions higher than 1000, log2FC differences between at least two experimental groups of 0.5–2.0. a qPCR results presented as mRNA expression changes from male and female vehicle podocytes (n = 4–8) in the left panel (P-values are indicated below each gene name), right panel shows expression level changes of RNA-seq for respective genes of vehicle groups, all genes were normalized to male vehicle, b qPCR results presented as mRNA expression changes from male rapamycin and male vehicle podocytes (n = 4–8) in the left panel (P-values are indicated below each gene name), right panel shows expression level changes of RNA-seq for respective genes of male rapamycin and vehicle groups, all genes were normalized to male vehicle (c) qPCR results presented as mRNA expression changes from female rapamycin and female vehicle podocytes (n = 5–9) in the left panel (P-values are indicated below each gene name), right panel shows expression level changes of RNA-seq for respective genes of female rapamycin and vehicle groups, all genes were normalized to female vehicle, Reference genes used for normalization were Hdgf and Ywhaz [file 13293_2024_647_MOESM4_ESM.pdf]
