# Supplementary material for: Sex-specific molecular signature of mouse podocytes in homeostasis and in response to pharmacological challenge with rapamycin
Source: Biol Sex Differ. 2024 Sep 15;15:72. doi: 10.1186/s13293-024-00647-7 (PMC11404044; doi:10.1186/s13293-024-00647-7)
Supplement: Supplementary file 5 — Additional file 5: Figure S4. Validation of proteomics data. a Western blots of selected intrinsically sexually dimorphic podocyte-specific proteins (left panel) and quantification (right panel) (n = 4 in each group) of proteins isolated from male and female podocyte-enriched kidney cortex tissue. All proteins were normalized to GAPDH. Results are expressed as mean ± SEM, Mann-Whitney test was used for statistical comparisons. b Relative differential expression levels of selected proteins as determined by proteomics and immunoblotting. [file 13293_2024_647_MOESM5_ESM.pdf]

Supplementary Figure S4

Proteomics data validation for selected podocyte-specific proteins

a Expression of podocyte-specific proteins in male and female renal cortex tissue

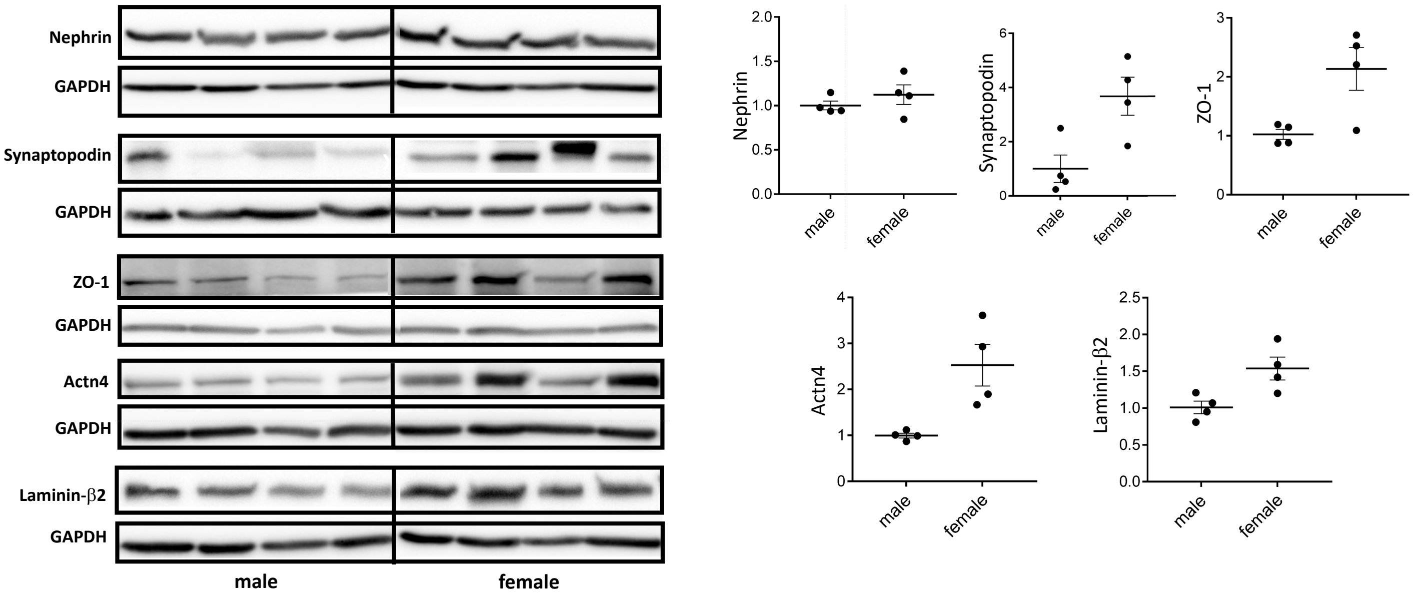

b Sex-differential expression of podocyte-specific proteins as determined by proteomics and immunoblotting

Protein name	FC male/female (proteomics)	FC male/female (WB)
Nephrin	0.86	0.85
Synaptopodin	0.43	0.28
ZO-1	0.48	0.454
Actn4	0.54	0.4
Lamininβ2	0.19	0.67
