# Supplementary material for: Sex-specific molecular signature of mouse podocytes in homeostasis and in response to pharmacological challenge with rapamycin
Source: Biol Sex Differ. 2024 Sep 15;15:72. doi: 10.1186/s13293-024-00647-7 (PMC11404044; doi:10.1186/s13293-024-00647-7)

Supplementary Figure S5

Functional groups of sexually dimorphic genes related to kidney diseases

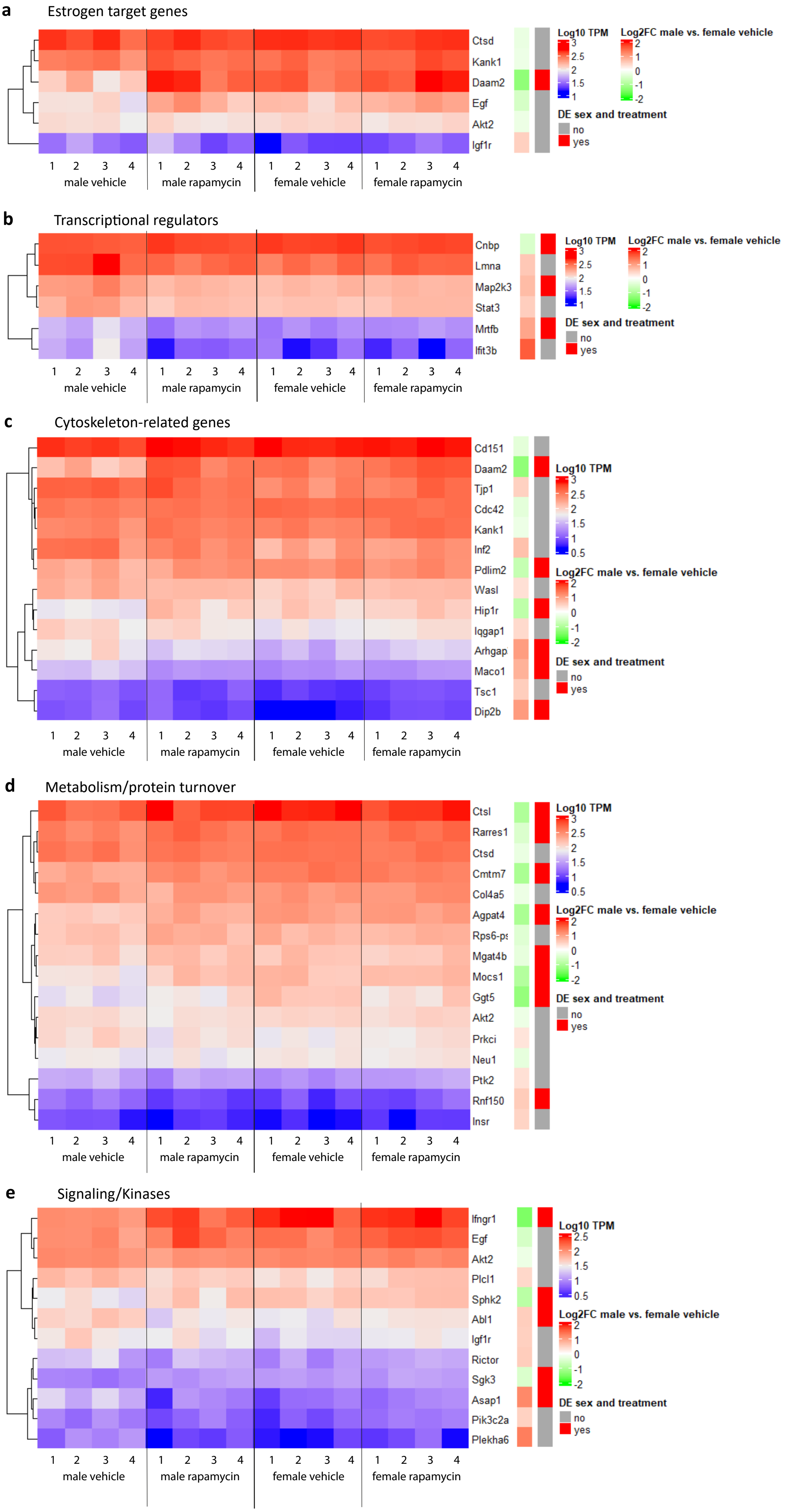

Supplement: Supplementary file 6 — Additional file 6: Figure S5. Functional grouping of intrinsic sexually dimorphic podocyte genes related to kidney diseases. Heatmap of the expression levels and log2FC of functional groups of sexually dimorphic genes related to kidney diseases. a estrogen target, b Transcriptional regulators, c Cytoskeleton-related genes, d metabolism and protein turnover and e signaling pathways/kinases related genes. Blue indicates decreased expression level, white no regulation, red increased gene expression level. (4 biological replicates in each group). [file 13293_2024_647_MOESM6_ESM.pdf]
