# Supplementary material for: Sex-specific molecular signature of mouse podocytes in homeostasis and in response to pharmacological challenge with rapamycin
Source: Biol Sex Differ. 2024 Sep 15;15:72. doi: 10.1186/s13293-024-00647-7 (PMC11404044; doi:10.1186/s13293-024-00647-7)

Supplementary Figure S7

a Intrinsic sex difference in AMPK activation levels

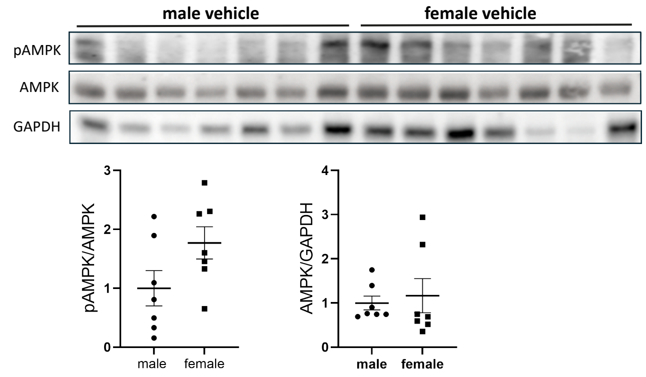

b Sexually dimorphic changes of AMPK activation levels in response to rapamycin

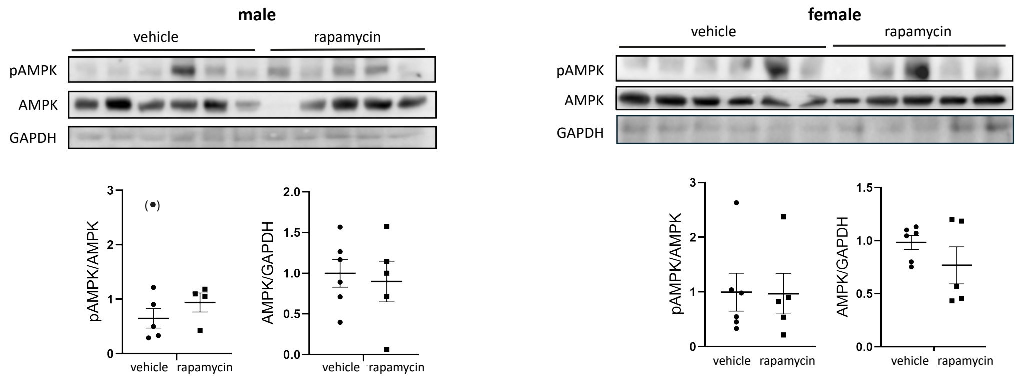

Supplement: Supplementary file 8 — Additional file 8: Figure S7. pAMPK and AMPK expression in male and female kidney cortex tissue. Western blots indicating activation levels of AMPK by phosphorylation of AMPK at Thr172 and AMPK expression in podocyte-enriched kidney cortex tissue with densitometric quantifications normalized to AMPK for pAMPK and GAPDH for AMPK for a male (n = 7) and female (n = 7) vehicle groups and b vehicle (n = 6) and rapamycin (n = 5) in male in the left panel and vehicle (n = 6) and rapamycin (n = 5) in female in the right panel. No statistical differences were detected when all biological replicates shown here were included in the analyses. However, when considering the 4th vehicle replicate in the male vehicle and rapamycin groups as an outlier, mean value of pAMPK/AMPK for vehicle was less compared to rapamycin as visualized in the scatter blot below the western blots. [file 13293_2024_647_MOESM8_ESM.pdf]
